# Selenium-modified graphene oxide: A tri-dimensional study of its cytotoxicity and developmental effects

**DOI:** 10.1016/j.mtbio.2025.102650

**Published:** 2025-12-08

**Authors:** Tuba Oz, Suresh K. Verma, Aleksey Kuznetsov, Palaniappan Nagarajan, Ivan Cole, Shaikh Sheeran Naser, Krzysztof Książek, Hong Yin, Małgorzata Kujawska

**Affiliations:** a Department of Toxicology, Poznan University of Medical Sciences, Poznan, Poland; b Doctoral School, Poznan University of Medical Sciences, Poznan, Poland; c School of Biotechnology, KIIT University, Bhubaneswar, Odisha, India; d Department of Chemistry, Universidad Técnica Federico Santa Maria, Campus Vitacura, Santiago, Chile; e School of Chemical Science, Central University of Gujarat, India; f School of Engineering, the Australian National University, Canberra, Australia; g Department of Pathophysiology of Ageing and Civilization Diseases, Poznan University of Medical Sciences, Poznan, Poland

**Keywords:** Graphene oxide, Selenium, Cytotoxicity, Oxidative stress, Zebrafish, Apoptosis, DFT study

## Abstract

Graphene oxide functionalized with selenium (GO-Se) has emerged as a promising nanomaterial due to selenium's antioxidant and redox-regulating properties, yet its safety profile remains unclear. The present study systematically investigated the cytotoxic, oxidative, and developmental effects of GO-Se using normal human dermal fibroblasts (NHDF) cells, zebrafish embryos and complementary density functional theory (DFT) calculations. GO-Se induced a dose- and time-dependent reduction in NHDF cell viability (IC_50_ = 274.6 μg mL^−1^ at 24 h), associated with oxidative stress modulation and apoptosis. Zebrafish models revealed concentration-dependent cardiac dysfunction, developmental abnormalities, and increased reactive oxygen species (ROS) levels at ≥100 μg mL^−1^. DFT analyses supported these findings by showing strong electron-accepting properties of GO-Se. Overall, the study highlights both the biomedical potential and the safety concerns of GO-Se, underlining the need for further investigations into its applicability.

## Highlights

• Selenium-modified graphene oxide (GO-Se, 100 nm) was synthesized as a novel nanomaterial.
• GO-Se reduced NHDF cell viability in a dose- and time-dependent manner (IC_₅₀_ = 274.6 μg mL^-1^, 24 h).
• Mechanistic assays revealed oxidative stress and apoptosis as key cytotoxic triggers.
• Zebrafish embryos exhibited toxic responses, including heart dysfunction and oxidative stress, at higher (≥100 μg mL^−1^) GO-Se concentrations.
• DFT calculations supported experimental findings and elucidated GO-Se toxicity mechanism.

## Graphical abstract

**Figure:**
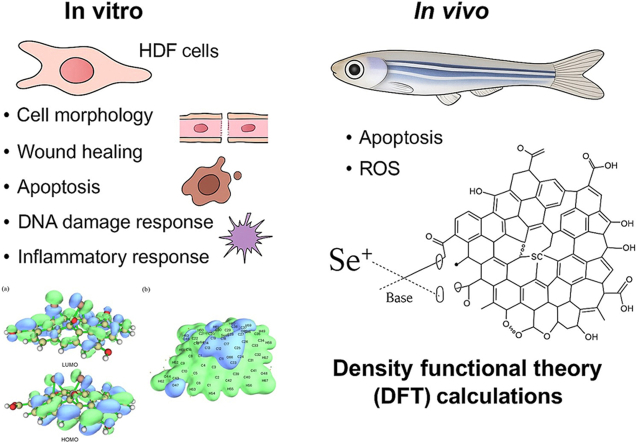

## 1 Introduction

Graphene-based nanomaterials (GBNs) have gained widespread attention for biomedical applications due to their high surface area, mechanical strength, and tunable functionalization. Among them, graphene oxide (GO) has been extensively studied for drug and gene delivery, biosensing, and regenerative medicine [[1], [2], [3]]. One emerging area of research focuses on the role of GBNs in modulating protein aggregation in neurodegenerative disorders, particularly synucleinopathies such as Parkinson's disease [4,5]. However, concerns regarding its cytotoxicity, genotoxicity, and biocompatibility have remained barriers to clinical translation [6,7]. The physicochemical properties of GO—including particle size, oxidation state, and surface chemistry—have been shown to significantly influence its cellular interactions and toxicity profiles [8,9]. Therefore, surface modification strategies have been explored to enhance GO's biocompatibility while preserving its beneficial properties. Bio-compatible reduction methods using plant extracts, as well as functionalization with heparin, polymers, and surfactants, along with sonochemical approaches, have been developed to reduce both toxicity and ROS generation, thereby making GO and rGO safer for various applications, particularly in the biomedical field [[10], [11], [12], [13], [14]].

Selenium (Se), an essential trace element with potent antioxidant and anti-inflammatory properties, has been investigated for its role in regulating oxidative stress and immune responses in nanomaterials [15]. Graphene oxide functionalized with selenium (GO-Se) has been proposed as a strategy to reduce GO-induced toxicity while enhancing stability and antioxidant activity. Se-functionalized nanomaterials have demonstrated redox-modulating properties, acting as antioxidants under oxidative stress and as pro-oxidants in reductive environments [16]. In this study, we hypothesize that GO-Se may confer dual benefits: (i) modulation of redox activity through the intrinsic antioxidant/pro-oxidant properties of Se, and (ii) improved biocompatibility and stability of the material. Se functions as a cofactor in antioxidant defense systems, notably glutathione peroxidases [17]. Se nanoparticles and GO-Se nanocomposites have demonstrated strong glutathione peroxidase-like catalytic activity, effectively neutralizing peroxides and reducing oxidative stress in cellular models [18,19]. Based on this rationale, GO-Se was expected to attenuate reactive oxygen species (ROS)-mediated cytotoxicity while retaining favorable physicochemical characteristics.

Several studies have shown that GO exposure induces ROS generation, mitochondrial dysfunction, and apoptosis in various cell types [20,21]. Zebrafish (*Danio rerio*) models have been widely employed to assess nanomaterial toxicity, providing insights into cardiotoxicity, neurodevelopmental changes, and morphological abnormalities [22,23]. Despite its potential benefits, the safety profile of GO-Se has not been fully established, particularly regarding its effects on fibroblasts and embryonic development.

In addition to experimental assessments, computational approaches such as density functional theory (DFT) provide valuable insights into the electronic structure, charge distribution, and molecular interactions of GO-Se, allowing for predictive modeling of its biological behavior and stability [19]. DFT studies have shown that GO-Se causes charge localization and modifies interfacial stability, which may influence its cytotoxicity, cellular uptake, and interactions with biomolecules [24]. These computational insights complement experimental toxicity assessments by elucidating the molecular-level interactions that govern GO-Se's biological behavior.

This study systematically evaluated the cytotoxicity and developmental toxicity of GO-Se using normal human dermal fibroblast (NHDF) cells and zebrafish embryos. We assessed its effects on cell viability, apoptosis, inflammatory cytokine modulation, and developmental abnormalities. Additionally, DFT calculations provided insights into GO-Se's electronic structure, charge distribution, and molecular interactions, elucidating its toxicity mechanisms. By integrating experimental and computational approaches, this study advanced our understanding of GO-Se's biological interactions and its potential biomedical applications.

## 2 Materials and methods

### 2.1 Synthesis and characterization of GO-Se

GO-Se was synthesized using a modified Hummers' method, followed by Se-functionalization through a reaction with sodium selenite under alkaline conditions. The final product was purified and characterized using X-ray diffraction (XRD) for crystallographic analysis, field emission scanning electron microscopy (FESEM) for morphological evaluation, transmission electron microscopy (TEM) for nanoscale structural insights, Fourier-transform infrared spectroscopy (FTIR) for chemical bonding analysis. These results have been previously published; here we provide a brief summary for clarity. TEM analysis indicated a sheet thickness of 50–80 nm, while FESEM/Energy-dispersive X-ray spectroscopy (EDX) mapping confirmed a uniform distribution of Se nanorods on GO surfaces. EDX confirmed the presence of Se signals, and X-ray photoelectron spectroscopy (XPS) revealed Se 3 d peaks consistent with Se(IV)/Se (0) oxidation states, thereby verifying successful Se incorporation [19]. The material displayed good colloidal stability, consistent with strong Se–GO interactions (Supplementary Data, Table S1). Also, the dynamic light scattering (DLS)-based size distribution histogram has been added (Supplementary Data, Fig. S1). The material was subsequently utilized for biological studies. For biological applications, a stock solution of GO-Se (1 mg mL^−1^) was prepared in serum-free medium and homogenized using an ultrasonic processor to ensure uniform dispersion. Then, samples were dispersed in this solution by ultrasonication for 10 min using a Sonoplus HD 4050 homogenizer (Bandelin, Germany) at 25 % of the maximum power.

### 2.2 *In vitro* studies

#### 2.2.1 Cell culture

NHDF cells (C-23210, PromoCell, Heidelberg, Germany) were cultured in DMEM (Sigma, D0819) supplemented with 10 % fetal bovine serum (FBS, Sigma, Lot: 0001,653,683), 1 μg mL^−1^ penicillin/streptomycin (Sigma, Lot: 0000191,002), and 2 mM L-Glutamine (Sigma, RNBL6712). Cells were maintained at 37 °C in a humidified atmosphere containing 5 % CO_2_ and sub-cultured at 80−90 % confluency.

#### 2.2.2 Measurement of *in vitro* cellular cytotoxicity

The cytotoxicity of GO-Se was evaluated using a 3-(4,5-dimethylthiazol-2-yl)-2,5-diphenyltetrazolium bromide (MTT) assay (Roche Cell Proliferation Kit I, Roche Diagnostics, Basel, Switzerland; cat. No. 11465007001) according to the manufacturer's protocol. NHDF cells between passages 4–6 were seeded in 96-well plates (5 × 10^3^ cells/well) and incubated at 37 °C with 5 % CO_2_ for 24 h. After adherence, cells were treated with GO-Se at concentrations ranging from 10 to 400 μg mL^−1^ for 24 h, 48 h, and 72 h. This broad range was applied to capture the full concentration–response relationship and to allow precise determination of the 50 % inhibitory concentration (IC_50_) values across different exposure times. Following treatment, 10 μL of MTT reagent (final concentration 0.5 mg mL^−1^) was added to each well, and plates were incubated for 4 h at 37 °C. Subsequently, 100 μL of solubilization buffer (10 % SDS in 0.01 M HCl) was added to each well, and plates were left overnight. Absorbance was measured at 570 nm using an ELISA reader (BioTek, USA).

#### 2.2.3 Cell morphology

NHDF cells were seeded in 6-well plates (2.5 × 10^5^ cells/well) and incubated at 37 °C with 5 % CO_2_ for 24 h. Cells were then treated with different doses of GO-Se for 24 h, 48 h, and 72 h. Morphological changes were observed using an inverted microscope (Nikon Eclipse TS100, USA).

#### 2.2.4 Apoptosis related cellular and nuclear morphology analysis

NHDF cells were seeded in 6-well plates (1 × 10^5^ cells/well) and treated with varying concentrations of GO-Se for 24 h, 48 h, and 72 h. After treatment, cells were stained with acridine orange (AO (ThermoScientific, cat. No. 300910250), 20 μg mL^−1^) and ethidium bromide (EtBr (E7637, Sigma), 1 mg mL^−1^) and incubated in the dark for 20 min. Excess dye was removed with phosphate-buffered saline (PBS), and apoptotic morphology was visualized using fluorescence microscope (Zeiss Axio Observer, USA).

#### 2.2.5 Measurement of intracellular ROS level

ROS levels were measured using 2′,7′-dichlorodihydrofluorescein diacetate (H_2_DCFDA) staining (D399; Molecular Probes, USA) following the manufacturer's protocol. NHDF cells were seeded in 6-well plates (1 × 10^5^ cells/well) and treated with GO-Se for 24 h, 48 h, and 72 h. Fluorescence microscopy included H_2_O_2_ as a positive control to demonstrate assay responsiveness, whereas flow-cytometry data were quantified relative to untreated controls. Cells were washed with PBS and stained with 10 μL of H_2_DCFDA. ROS levels were analyzed using a Cytek Amnis CellStream flow cytometer (Luminex Corporation) and a fluorescence microscope (Zeiss Axio Observer, USA). Data were processed using FlowJo™ software v10.10.0 (TreeStar, Ashland, OR, USA) and Prism 10 software (GraphPad Software, Inc., La Jolla, CA, USA).

#### 2.2.6 Biochemical assays

NHDF cells were seeded in 6-well plates at a density of 1 × 10^5^ cells/well and treated with GO-Se for 24 h, 48 h, and 72 h. After treatment, cells were washed twice with PBS, and harvested by centrifugation at 10,000×*g* for 20 min at 4 °C. The resulting pellets were resuspended in 1 mL of cold PBS and sonicated in short pulses on ice. The homogenates were centrifuged at 10,000×*g* for 15 min at 4 °C, and the resulting supernatants were collected and kept on ice for further analysis of catalase (CAT) and glutathione peroxidase (GPx) activities. Total protein concentration in the lysates was determined using the Bicinchoninic Acid Protein Assay Kit (Sigma; BCA1, B9643). CAT and GPx activities were measured spectrophotometrically, following the method described by Kujawska et al. (2014) [25]. CAT activity was assessed by monitoring the decomposition rate of hydrogen peroxide at 240 nm. GPx activity was determined according to Mohandas et al. (1984), based on the Nicotinamide Adenine Dinucleotide Phosphate (NADPH) oxidation rate, measured as a decrease in absorbance at 340 nm in the presence of hydrogen peroxide as the substrate [26]. Enzyme activities were normalized to the total protein content of the lysates.

#### 2.2.7 Cell migration

NHDF cells were seeded in 24-well plates (2 × 10^5^ cells/well) and incubated for 24 h. Once cells reached 100 % confluency, wounds were created using a 1 mL micropipette tip. Cells were treated with 275 μg mL^−1^ GO-Se, and migration was observed over time using an inverted microscope (Nikon Eclipse TS100, USA).

#### 2.2.8 DNA damage response antibody array

The expression of 27 DNA damage response (DDR)-associated proteins was analyzed using the RayBio® C-Series Human DNA Damage Response Antibody Array 1 (RayBiotech Life, USA) following the manufacturer's protocol. NHDF cells were seeded in 6-well plates (1 × 10^6^ cells/well) and treated with a cytotoxic dose of GO-Se for 24 h. After treatment, cells were lysed with ice-cold buffer containing a protease inhibitor cocktail. Lysates were centrifuged at 14,000×*g* for 5 min at 4 °C, and protein concentrations were determined using a Bicinchoninic Acid Protein Assay Kit (Sigma; BCA1, B9643). Arrays were incubated with 200 μg of total protein overnight at 4 °C, followed by biotinylated antibody incubation, horseradish peroxidase-streptavidin labeling, and chemiluminescence detection using an iBright Imaging System (Invitrogen). Densitometry analysis was performed using ImageJ software (NIH, Bethesda, MD, USA), and results were normalized relative to the positive controls.

#### 2.2.9 Inflammation array

The concentration of 40 cytokine proteins was quantified using the Human Inflammation Array 3 Kit (QAH-INF-3, RayBiotech, USA) following the manufacturer's instructions. NHDF cells were seeded in 6-well plates (1 × 10^6^ cells/well) and incubated for 24 h. After treatment, culture media were collected and diluted 2-fold in sample diluent. Arrays were blocked with sample diluent, incubated with 100 μL of samples overnight at 4 °C, followed by Cy3-streptavidin labeling, and scanned using a laser scanner. Data extraction was performed using the GAL file (www.RayBiotech.com/Gal-Files.html) and analyzed with GenePix software.

#### 2.2.10 Spheroid culture assay

For three-dimensional (3D) culture, agarose was employed as a biomimetic scaffold to simulate the *in vivo* microenvironment of NHDF cells. The agarose gel matrix (1.5 % w v^−1^) was prepared according to the protocol described by Le et al. (2024) [27]. Briefly, agarose powder (Sigma-Aldrich, A9539) was dissolved in PBS by heating the suspension until complete dissolution was achieved. The molten agarose was transferred into 1.5 mL centrifuge tubes and stored at 4 °C for future use. When required, the solidified agarose was remelted by incubation in boiling water for 5 min. A volume of 200 μL of hot agarose was then dispensed into the bottom of each well of a 24-well plate to form a non-adhesive surface. The plates were left at room temperature for approximately 30 min to allow complete gelation. NHDF cells were seeded onto the agarose-coated wells at a density of 2.5 × 10^5^ cells/well and cultured for 24 h. After this initial incubation, the cells were treated with GO-Se at a concentration of 275 μg mL^−1^ (the IC_50_ value for 24 h exposure) and maintained under standard culture conditions (37 °C, 5 % CO_2_) for up to 168 h. Spheroid formation was monitored over time using an inverted phase-contrast microscope (Nikon Eclipse TS100, USA).

### 2.3 *In vivo* studies

#### 2.3.1 Zebrafish maintenance and breeding

The adult zebrafish were reared and maintained in an overflow container system provided by Aquaneering, USA. The system was equilibrated with fish water comprising 75 g of NaHCO_3_, 18 g of sea salt, and 8.4 g of CaSO_4_ per 1000 mL. The fish were fed bloodworm-infused diet three times daily. The photoperiodism was adjusted to 12 h of darkness and 12 h of light, respectively. The embryos were acquired by culture and breeding in a system with a net partition, utilizing a female-to-male ratio of 2:1. The viable embryos were separated from the non-viable ones following their acquisition, followed by washing with sterilized Holtfreter buffer (HF; NaCl 59 mM, KCl 0.67 mM, CaCl_2_ 0.9 mM, NaHCO_3_ 2.4 mM; pH ∼7.4) media. All tests were conducted in filter-sterilized HF medium (pH 7.2, I ∼ 7.2 × 10^−2^ M), including 3.5 g of NaCl (Merck, Germany), 0.2 g of NaHCO_3_ (Merck, Germany), 0.05 g of KCl (Merck, Germany), and 0.12 g of CaCl_2_·2H_2_O (Himedia, India) per 1 L of aqueous solution. Reagents utilized for buffer preparation were acquired from Merck. All animal procedures were approved by the Institutional Animal Ethics Committee (IAEC) of KIIT Deemed to be University (Approval No: KSBTT/IAEC/2024/MEET-2/#16–3, dated December 10, 2024) and adhered to the pertinent criteria established by the Committee. All studies were conducted in accordance with the relevant animal welfare rules and regulations of the IAEC, KIIT University.

#### 2.3.2 Biocompatibility assessment

The biocompatibility evaluation of GO-Se was conducted utilizing 3–4 h post-fertilization (hpf) embryos at the blastula stage. 20 Embryos were exposed to a 24-well plate containing HF buffer with varying concentrations (25, 50, 100, 150, and 200 μg mL^−1^) of GO-Se for 72 h. After the experimental setup, the plate was incubated under a 12 h light/dark cycle at 28 ± 1 °C. Unexposed embryos served as the control group. Morphological alterations were identified and documented using a stereomicroscope. The mortality rate was assessed as the percentage ratio of viable to non-viable embryos at 72 hpf. The hatching rate was determined by comparing the number of embryos that hatched within 72 hpf to the unexposed group. The heartbeat rate was assessed as the number of beats per minute [23].

#### 2.3.3 Oxidative stress analysis

Oxidative stress assay in embryos treated with GO-Se was conducted by staining the embryos with 1.25 mg L^−1^ H_2_DCFDA dye for 20 min in darkness. After staining, the excess stain was eliminated by rinsing with HF buffer. Imaging was conducted with an EVOS inverted fluorescence microscope (ThermoScientific, USA). The quantification of the fluorescence was performed by using Image J [28].

#### 2.3.4 Assessment of apoptosis

The apoptosis of embryos was evaluated after AO staining. Zebrafish embryos, both unexposed and exposed to GO-Se, were stained with 5 μg mL^−1^ AO dissolved in HF buffer for 20 min. The excessive stain was removed via washing with HF buffer. Imaging was conducted using the green channel of the EVOS inverted fluorescence microscope (ThermoScientific, USA). The quantification of the fluorescence was performed by using Image J [28].

### 2.4 DFT study

DFT studies were conducted to provide the molecular-level insights into the electronic properties, charge distribution, and interactions with biological molecules, complementing the experimental cytotoxicity and biocompatibility assessments. The studies were performed with the Gaussian 16 [29] software using the hybrid density functional B3LYP [30] and the double-zeta split-valence polarized basis set 6-31G∗ (one set of polarization functions) [31,32]. This approach is referred to as B3LYP/6-31G∗. We chose the model for the Se-modified reduced graphene oxide (RGO-Se) with the stoichiometry C_43_H_16_O_6_Se_2_. The RGO-Se contains one CO_2_H- and two OH-groups attached to the edge of the graphene model. This RGO model was chosen to make our calculations more facile, because the GO model with numerous oxygen-containing groups would demand more computational resources for its studies, which we did not have. Typical GO defects were not included, for the simplicity sake, but some edge groups like hydroxyls and carboxyl were included. Both singlet and triplet structures were studied. Geometry optimization and frequency calculations were performed with the B3LYP/6-31G∗ approach in the gas phase or considering the implicit effects from water (dielectric constant ε = 78.3553). A self-reliable integral-equation-formalism polarizable continuum model (IEF-PCM) approach was employed [33] with the universal force field (UFF) default model as implemented in the Gaussian 16 software, and the electrostatic scaling factor α was set to 1.0.

Below we consider the results of natural bond orbital (NBO) analysis [34] and frontier molecular orbitals (FMOs) for the global minimum singlet structure of RGO-Se. Furthermore, we use the highest occupied molecular orbital (HOMO) and the lowest unoccupied molecular orbital (LUMO) energies to compute its global reactivity parameters (GRPs) [35,36] (Supplementary Data, Equations). Furthermore, we performed molecular electrostatic potential (MEP) [37] and quantum theory atom-in-molecule (QTAIM) [38] analyses. GO-Se molecular structure, FMOs, and MEP plots were visualized using Avogadro visualization software (version 1.1.1) [39]. Gabedit (version 2.5.1), a graphical user interface for computational chemistry software, was used to calculate the GRP values [40]. Multiwfn software was used for the QTAIM analysis [41].

### 2.5 Statistical analysis

All statistical analyses were conducted for *in vitro* experiments using Prism 10 software (GraphPad Software, Inc., La Jolla, CA, USA). Data distributions were assessed for normality and log-normality using the D'Agostino & Pearson omnibus test, Anderson–Darling test, Shapiro–Wilk test, and Kolmogorov–Smirnov test, with a significance threshold of α = 0.05. Based on the distribution characteristics, appropriate statistical tests were applied. For comparisons between two groups, the unpaired Student's t-test was used for normally or log-normally distributed data, while the Mann–Whitney *U* test was applied when normality assumptions were not met. Comparisons among three or more groups were conducted using one-way ANOVA followed by Tukey's or Sidak's multiple comparison test when parametric assumptions were fulfilled. If the data did not meet these assumptions, the Kruskal–Wallis test followed by Dunn's post hoc test was used. The IC_50_ values were calculated using non-linear regression analysis, and heat maps were generated in Prism 10. Data are presented as mean ± standard deviation (SD) or median ± interquartile range (IQR), as specified in the figure legends.

Statistical analysis of data related to embryonic zebrafish cytotoxicity assessment was done using GraphPad Prism v9 (GraphPad Software, Inc., La Jolla, CA, USA). Respective confidential intervals were determined by the non-linear fit of the sigmoidal dose response curve. Normality of data distributions was assessed prior to statistical testing. Data were analyzed by one way ANOVA followed by Tukey/*t*-test with significance set at p < 0.05. Data are presented as mean ± SD.

## 3 Results

### 3.1 Effect of GO-Se on cell viability

**Fig. 1:**
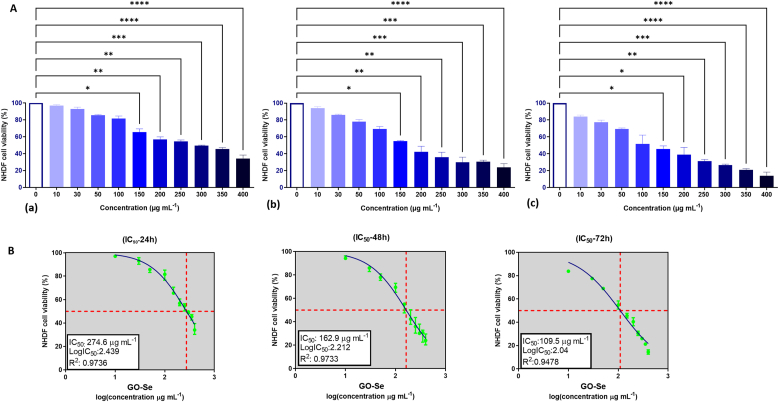
(A) Viability of NHDF cells exposed to different concentrations of GO-Se as determined by MTT assay. (a); 24 h, (b); 48 h, (c); 72 h. Control cells were assigned 100 % viability. The experiments were conducted in quadruplicate (n = 4), and the data are expressed as median ± IQR. ∗p < 0.05, ∗∗p < 0.01, ∗∗∗p < 0.001, ∗∗∗∗p < 0.0001 by Kruskal–Wallis test with Dunn's Multiple comparison test. (B) IC_50_ of GO-Se in NHDF cells at 24 h, 48 h, and 72 h.

To assess the cytotoxic effects of GO-Se, NHDF cells were treated with increasing concentrations of GO-Se (0–400 μg mL^−1^), and cell viability was evaluated using the MTT assay at 24 h, 48 h, and 72 h. The results revealed a dose- and time-dependent decrease in NHDF cell viability upon GO-Se exposure. At all time points, a progressive reduction in cell viability was observed with increasing GO-Se concentrations, with significant cytotoxicity detected at concentrations ≥150 μg mL^−1^ (Fig. 1A). The IC_50_ values of GO-Se were calculated for each time point to quantify its cytotoxic potential. The IC_50_ values were 274.6, 162.9, and 109.5 μg mL^−1^ at 24 h, 48 h, and 72 h, respectively, demonstrating a progressive increase in cytotoxicity over time (Fig. 1B). This trend indicates that NHDF cells exhibit increasing sensitivity to GO-Se with prolonged exposure, reinforcing the time-dependent nature of its cytotoxic effects.

### 3.2 Effect of GO-Se on cell morphology

To investigate whether GO-Se-induced cytotoxicity was associated with morphological alterations and apoptosis, NHDF cells were examined under an inverted microscope. Control cells maintained a typical spindle-like morphology, whereas GO-Se-treated cells exhibited progressive morphological changes, including cell rounding, detachment, and cytoplasmic shrinkage (Supplementary Data, Fig. S2). These changes became more pronounced with the increases in concentration and exposure time, consistent with apoptotic cell death.

### 3.3 Effect of GO-Se on apoptotic morphological changes

Fluorescence microscopy was employed to assess whether the cytotoxic effect of GO-Se is associated with apoptosis induction. In viable cells, the nuclei appeared uniformly bright green, whereas early apoptotic cells exhibited bright green areas of fragmented chromatin. Late apoptotic or necrotic cells displayed a brightly orange-stained nucleus, indicative of compromised membrane integrity.

**Fig. 2:**
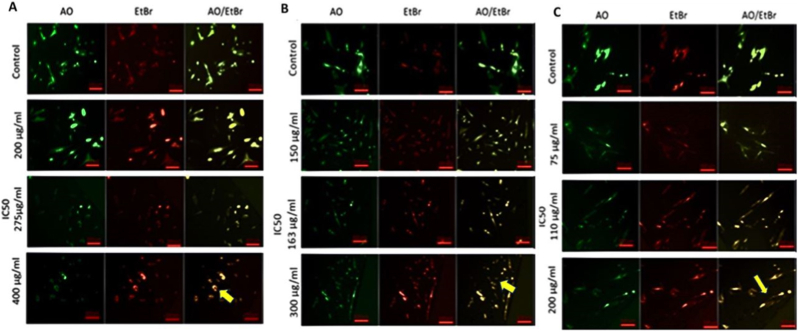
Panels **A**, **B**, and **C** depict apoptotic morphological changes observed at 24 h, 48 h and 72 h, respectively, using AO/EtBr staining and fluorescence microscopy (10 × ). NHDF cells were treated with GO-Se at sub-IC_50_ (200 μg mL^−1^ for 24 h, 150 μg mL^−1^ for 48 h, and 75 μg mL^−1^ for 72 h), IC_50_ (275 μg mL^−1^ for 24 h, 163 μg mL^−1^ for 48 h, and 110 μg mL^−1^ for 72 h), and supra-IC_50_ (400 μg mL^−1^ for 24 h, 300 μg mL^−1^ for 48 h, and 200 μg mL^−1^ for 72 h) doses at each respective time point. Data represent three independent experiments (n = 3). The yellow arrows represented cells exposed to supra-IC_50_ concentrations exhibited extensive nuclear condensation, chromatin fragmentation, and apoptotic body formation. Scale bar: 200 μm. (For interpretation of the references to color in this figure legend, the reader is referred to the Web version of this article.)

Cells exposed to GO-Se at concentrations below, at, and above the IC_50_ dose for 24 h (275 μg mL^−1^), 48 h (163 μg mL^−1^), and 72 h (110 μg mL^−1^) exhibited nuclear condensation, chromatin fragmentation, and apoptotic body formation in a dose- and time-dependent manner. Notably, cells treated at doses lower than IC_50_ showed mild chromatin condensation, whereas those exposed to concentrations above IC_50_ displayed extensive chromatin fragmentation and late apoptotic characteristics, such as nuclear shrinkage and membrane blebbing (Fig. 2).

### 3.4 Effect of GO-Se on intracellular ROS level

**Fig. 3:**
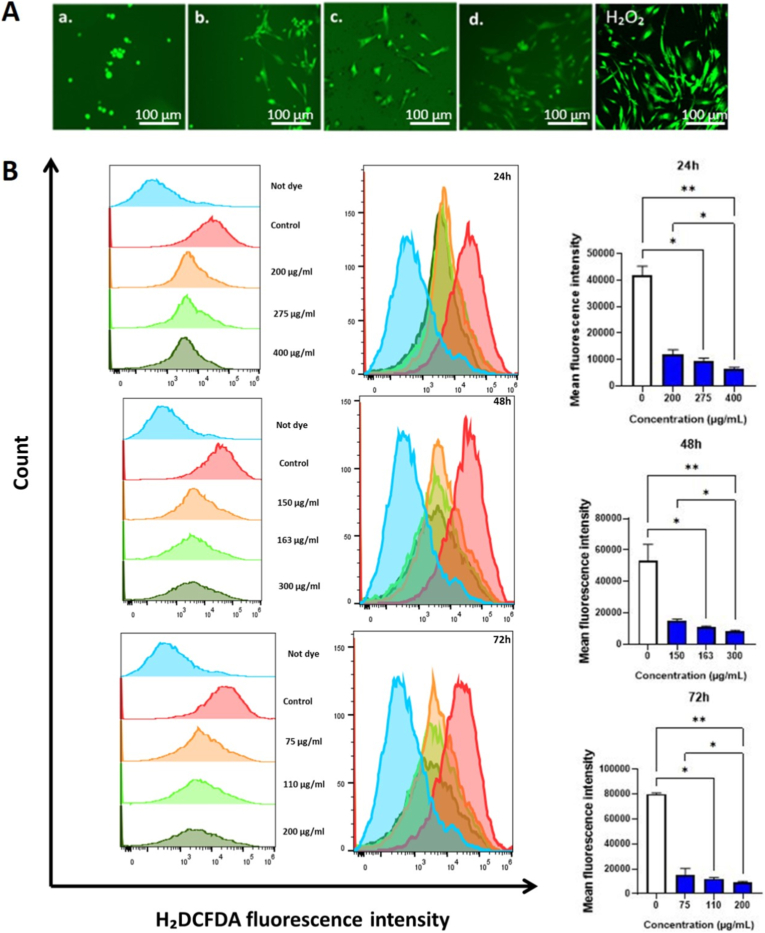
**(A)** Fluorescence microscopy images (10 × ) showing ROS levels in control and GO-Se-treated NHDF cells at the IC_50_ dose after 24 h, 48 h, and 72 h. a) Control, b) 24 h: IC_50_ dose 275 μg mL^−1^, c) 48 h: IC_50_ dose 163 μg mL^−1^, and d) 72 h: IC_50_ dose 110 μg mL^−1^. **H_2_O_2_:** positive control. Scale bar, 100 μm. **(B)** Flow cytometry analysis of ROS production in NHDF cells treated with GO-Se for 24 h, 48 h, and 72 h. Flow cytometry analysis of ROS production was normalized to untreated controls. Assay responsiveness was confirmed in the microscopy arm with H_2_O_2_ as a positive control. The bar graph presents the quantification of H_2_DCFDA fluorescence intensity in NHDF cells treated with GO-Se. Data are expressed as median ± IQR from three independent (n = 3) experiments by one-way ANOVA followed by Tukey's multiple comparisons test: ∗p < 0.05, ∗∗p < 0.01 vs. control.

The mechanisms underlying the impact of GO-Se on cells was further investigated by determining its ability to induce oxidative stress. NHDF cells were treated with different concentrations of GO-Se for 24 h, 48 h, and 72 h and stained with H_2_DCFDA to evaluate intracellular ROS induction. Fluorescence microscopy included an H_2_O_2_ positive control to demonstrate assay responsiveness, whereas flow-cytometry measurements were normalized to the untreated control, providing an internally consistent reference across time points. Flow cytometry analysis (Fig. 3A) revealed a dose-dependent suppression of ROS, with significant reductions observed at higher GO-Se concentrations at all three time points. Fluorescence intensity quantification (Fig. 3B) further confirmed that GO-Se-treated cells exhibited lower ROS levels compared to untreated controls. These findings suggest that GO-Se effectively reduces intracellular ROS levels in NHDF cells, possibly contributing to oxidative stress modulation.

### 3.5 Effect on GO-Se on activity of CAT and GPx

The activity of CAT and GPx were analyzed for the NHDF cells treated with different GO-Se concentrations for 24, 48, and 72 h.

**Fig. 4:**
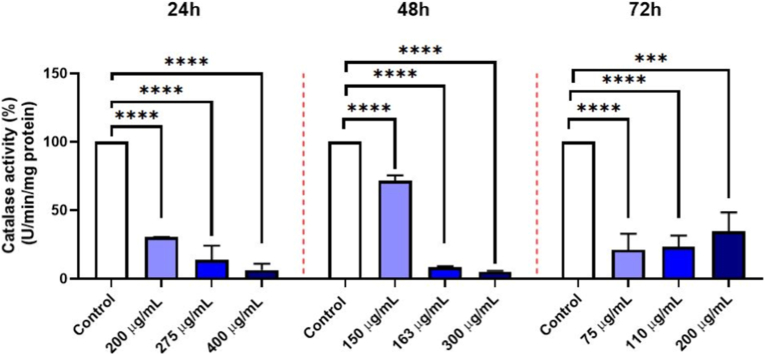
Effect of GO-Se on CAT activity (% of control) in NHDF cells after 24 h, 48 h and 72 h treatment. Data are presented as mean ± SD from three independent experiments (n = 3). Statistical significance was determined using one-way ANOVA followed by Tukey's multiple comparisons test: ∗∗∗p < 0.001, ∗∗∗∗p < 0.0001 vs. control.

After 24 h, CAT activity in cells treated with GO-Se at 200, 275, and 400 μg mL^−1^ was significantly inhibited by 70 %, 86 %, and 93 %, respectively, compared to the control, (p < 0.0001). At 48 h, CAT activity was reduced by 30 %, 91 %, and 94 % following treatment with GO-Se at 150, 163, and 300 μg mL^−1^, respectively (p < 0.0001). After 72 h, CAT activity was suppressed by 86 %, 83 %, and 77 % in cells treated with GO-Se at 75, 110, and 200 μg mL^−1^, respectively (p < 0.001) (Fig. 4).

**Fig. 5:**
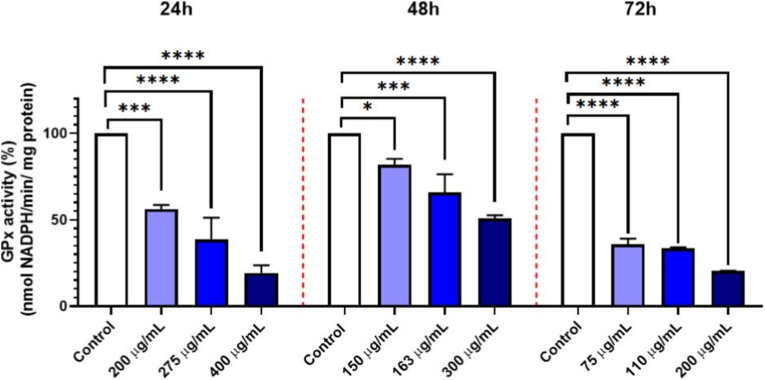
Effect of GO-Se on GPx activity (% of control) in NHDF cells after 24 h, 48 h, and 72 h of treatment. Data are presented as mean ± SD from three independent experiments (n = 3). Statistical significance was determined using one-way ANOVA followed by Tukey's multiple comparisons test: ∗p < 0.05, ∗∗∗p < 0.001, ∗∗∗∗p < 0.0001 vs. control.

Similarly, GPx activity was inhibited in NHDF cells treated with GO-Se at 200, 275, and 400 μg mL^−1^ for 24 h, resulting in reductions of 39 %, 62 %, and 86 %, respectively. At 48 h, GPx inhibition was observed at 150, 163, and 300 μg mL^−1^, with reductions of 18 %, 31 %, and 49 %, respectively. After 72 h, cells treated with 75, 110, and 200 μg mL^−1^ showed GPx activity decreases of 64 %, 67 %, and 80 %, respectively (Fig. 5).

The inhibitory effect of GO-Se on antioxidant enzymes was clearly concentration-dependent. CAT activity showed progressive suppression across all exposure times, with the strongest inhibition observed at supra-IC_50_ concentrations. A similar pattern was detected for GPx, where enzyme activity decreased proportionally to the GO-Se dose at 24 h, 48 h, and 72 h. These results indicate that GO-Se consistently suppresses antioxidant defenses in NHDF cells in a dose-dependent manner, which may contribute to the observed redox imbalance and cytotoxicity.

### 3.6 Effect of GO-Se on cell migration

**Fig. 6:**
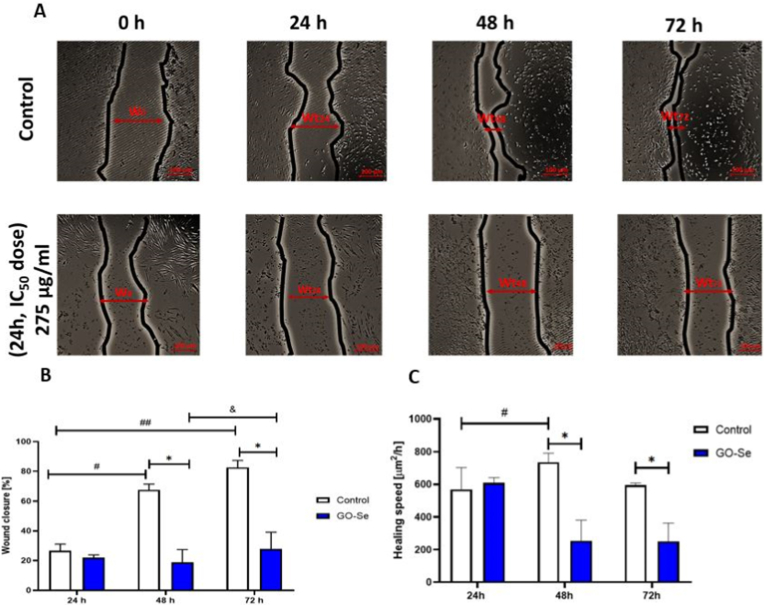
**(A)** An *in vitro* wound healing model was used to examine the effect of GO-Se on NHDF cell migration. W_0_: wound area at 0 h (μm^2^). Wt_24_, Wt_48_, and Wt_72_: wound area at 24 h, 48 h, and 72 h, respectively. Scale bar, 100 μm. **(B)** Quantification of wound closure at 24 h, 48 h, and 72 h in control and GO-Se (275 μg mL^−1^)-treated NHDF cells, presented as relative units (%). Results represent the mean of four measurements per wound area, based on four independent experiments (n = 4) by Kruskal–Wallis test and Dunn's Multiple comparison test. # and ## indicate comparisons between control groups at different time points (p < 0.05; p < 0.01). & indicates comparisons between GO-Se (275 μg mL^−1^)-treated NHDF cells at 48 h and 72 h (p < 0.05). ∗ indicates comparisons between the control and GO-Se (275 μg mL^−1^)-treated NHDF cells at 48 h and 72 h. **(C)** Quantification of wound healing speed (μm^2^ h^−1^) at 24 h, 48 h, and 72 h in control and GO-Se (275 μg mL^−1^)-treated NHDF cells**.** # Indicates comparisons between control groups at 24 h and 48 h (p < 0.05); ∗ indicates comparisons between control and GO-Se (275 μg mL^−1^)-treated NHDF cells at 48 h and 72 h (p < 0.05).

Fibroblast migration is a fundamental cellular function, and its impairment is considered an indicator of cytotoxicity and compromised biocompatibility; therefore, the wound healing assay was performed to assess whether GO-Se alters fibroblast motility in addition to its effects on viability and apoptosis [42]. To evaluate the effect of GO-Se on fibroblast migration, an *in vitro* wound healing assay was performed. NHDF cells were treated with GO-Se (275 μg mL^−1^, IC_50_ dose for 24 h), and wound closure was assessed at 24 h, 48 h, and 72 h (Fig. 6A).

Wound closure (%) demonstrated a time-dependent increase in migration in both the control and GO-Se-treated groups. In the control group, a significant increase in wound closure was observed between 24 h and 48 h, and this increase was maintained at 72 h (p < 0.05). In contrast, in the GO-Se-treated group, a significant difference was detected only between 48 h and 72 h (p < 0.05), indicating a delayed progression of wound closure. However, GO-Se-treated cells exhibited significantly lower wound closure at 48 h and 72 h, compared to the control group (p < 0.05), confirming a persistent delay in wound healing (Fig. 6B). Analysis of wound healing speed (μm^2^ h^−1^) revealed a significant increase between 24 h and 48 h in the control group (p < 0.05), with no further increase between 48 h and 72 h. In contrast, no significant changes in healing speed were detected at any time point in the GO-Se-treated group, indicating a sustained reduction in fibroblast motility throughout the experiment (Fig. 6C).

### 3.7 Effect of GO-Se on DDR

**Fig. 7:**
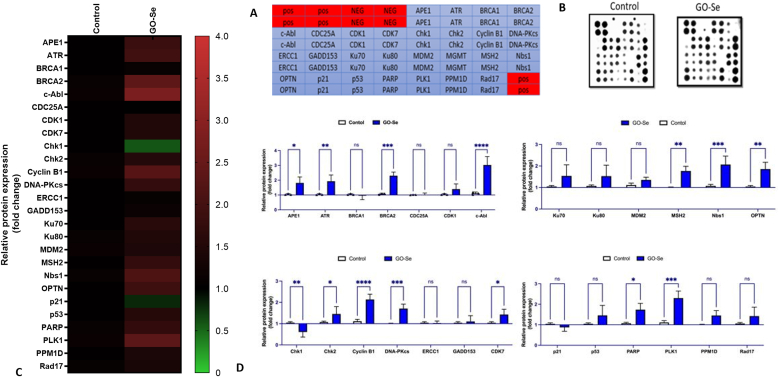
Expression profiles of DDR-related proteins in NHDF cells following 24 h exposure to GO-Se at 275 μg mL^−1^ (IC_50_). **(A)** Antibody array layout showing antigen-specific antibody spots; “nbs1” control spots were used for data normalization, and “NEG” spots served as negative controls for baseline signal measurement. **(B)** Representative images of the original antibody arrays. **(C)** Heat map illustrating the relative expression levels of DDR-related proteins, with color intensity indicating normalized expression values. Data represent four independent experiments (n = 4). **(D)** Semi-quantitative analysis of DDR-related proteins expression using antibody microarray in NHDF cells treated with GO-Se at 275 μg mL^−1^ for 24 h. Data are expressed as mean ± SD (n = 4) relative to control cells. Statistical significance was assessed using two-way ANOVA, followed by Sidak's multiple comparisons test: ns: not significant, ∗p < 0.05, ∗∗p < 0.01, ∗∗∗p < 0.001, ∗∗∗∗p < 0.0001 vs. control. (For interpretation of the references to color in this figure legend, the reader is referred to the Web version of this article.)

Given GO-Se's ability to modulate oxidative stress and influence cellular viability, its impact on genomic stability was evaluated by assessing the DDR in NHDF cells. The expression profiles of 27 proteins involved in DDR were monitored in NHDF cells using antibody arrays (Supplementary Data; Table S2, and Fig. 7). The semi-quantitative analysis of protein expression revealed that GO-Se treatment significantly altered the expression of key proteins involved in homologous recombination (HR) repair, base excision repair (BER), and cell cycle regulation. Specifically, *APE1, ATR, BRCA2, c-Abl, Chk2, Cyclin B1, DNA-PKcs, MSH2, NBS1, OPTN, PARP,* and *PLK1* were upregulated while *Chk1* and *p21* were downregulated (Fig. 7D). This suggests that GO-Se exposure triggers an adaptive response in NHDF cells, potentially favoring an alternative DNA repair mechanism over the canonical HR repair pathway. Therefore, the DNA-damaging activity plays a critical role in cell cytotoxicity.

### 3.8 Effect of GO-Se on inflammation

**Fig. 8:**
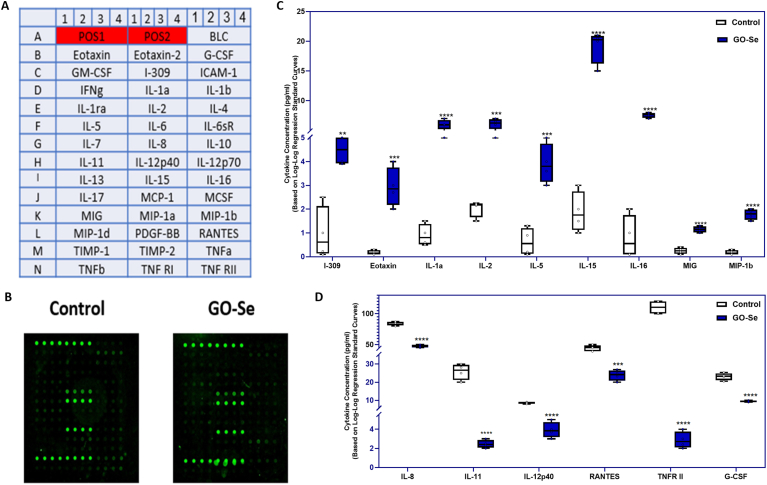
Expression profiles of cytokine-related factors in NHDF cells following 24 h treatment with GO-Se at 275 μg mL^−1^. A human cytokine antibody array containing 40 cytokines was used, with “POS” positive control spots applied for data normalization. **(A)** Each antibody was spotted in quadruplicate in a horizontal layout. **(B)** Representative fluorescence images of the cytokine antibody arrays. **(C)** Upregulated cytokines identified in NHDF cells treated with GO-Se (275 μg mL^−1^, 24 h). The array detected nine significantly upregulated cytokines compared to control cells, including chemokines, metalloproteinase, interleukins/receptors, and macrophage inflammatory protein. **(D)** Downregulated cytokines identified in GO-Se–treated NHDF cells. Six cytokines were significantly downregulated, comprising adhesion receptor, metalloproteinase, interleukins/, macrophage inflammatory protein, and colony-stimulating factor. Data represent four independent experiments (n = 4). Statistical analysis was performed using an unpaired Student's t-test. ∗∗p < 0.01, ∗∗∗p < 0.005, ∗∗∗∗p < 0.0001 vs. control.

Human cytokine microarrays were employed to detect proteins in control and NHDF cells treated with GO-Se. In the screening stage, all 40 proteins (Supplementary Data; Table S3, and Fig. 8A) could be detected in QAH-INF-3 by building a standard curve with a serial dilution of standard samples, and the fluorescence image is shown in Fig. 8B. As shown in Fig. 8C, nine cytokines (*I-309, Eotaxin, IL-1α, IL-2, IL-5, IL-15, IL-16, MIG, and MIP-1b*) were significantly upregulated in GO-Se–treated NHDF cells compared to untreated control cells. In contrast, six cytokines (*IL-8, IL-11, IL-12p40, RANTES, TNFR II, G-CSF*) were significantly downregulated (Fig. 8D). These findings are based on densitometric analysis normalized to positive controls, and the changes are statistically significant (p < 0.05). The altered profile suggests that GO-Se modulates fibroblast immune signaling, with the upregulation of key chemokines, interleukins, and matrix metalloproteinase inhibitors.

### 3.9 *In vivo* toxicity evaluation of GO-Se in embryonic zebrafish

**Fig. 9:**
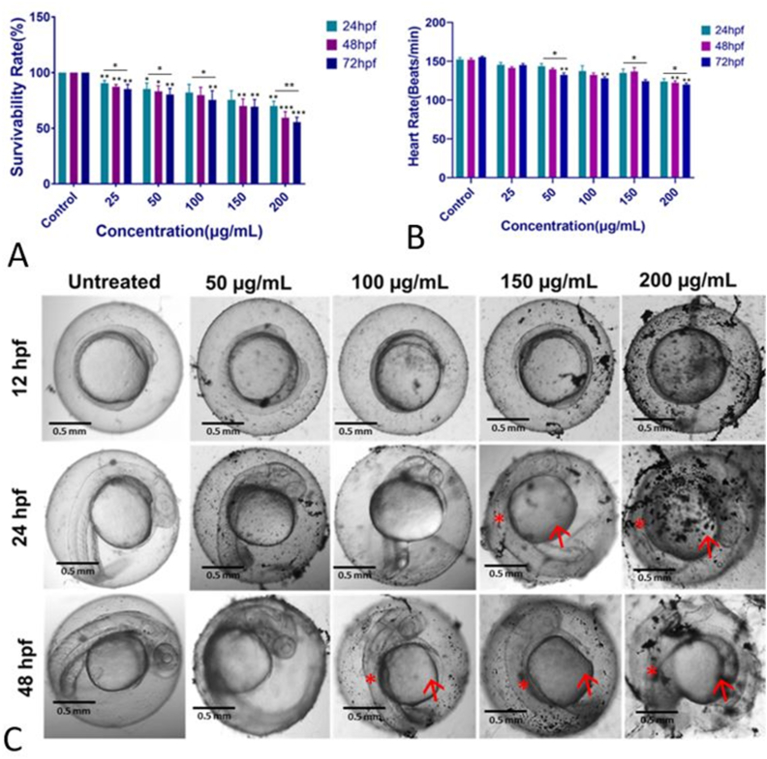
*In vivo* physiological toxicological effect of GO-Se on embryonic zebrafish; **(A)** Survivability rate of the embryos exposed to different concentrations of GO-Se. **(B)** Heart rate of the embryos exposed to different concentrations of GO-Se. The values are presented as mean ± SD from 20 embryos [23]. All the experimental analyses were done in triplicate and thrice independently. ∗p > 0.05, ∗∗p > 0.01, and ∗∗∗p > 0.001 denote the compared significant change at each exposed concentration compared to untreated embryos as obtained from post hoc analysis after one-way ANOVA. **(C)***In vivo* toxicological effect of GO-Se on embryonic zebrafish; morphological abnormalities in zebrafish embryos exposed to different concentrations of GO-Se. (→Pericarfial edema, ∗ Abnormal notochord).

The *in vivo* toxicity of GO-Se was analyzed by evaluating its effect on the developmental stage using an embryonic model. As shown in Fig. 9, the survivability of the embryos was found to be time- and dose-dependent on the GO-Se exposure. Interestingly, survivability was found to decrease with an increased exposure concentration of GO-Se (Fig. 9A). The lethal concentration 50 % (LC_50_) value of GO-Se was calculated as 436.7 ± 5.0 μg mL^−1^, 316.6 ± 5.0 μg mL^−1^, and 273.0 ± 5.0 μg mL^−1^ for 24 hpf, 48 hpf, and 72 hpf embryos (Supplementary Data, Fig. S4). In addition, the heartbeat rate was measured to assess the cellular effect, and it was found to decrease with an increase in exposure concentration of GO-Se (Fig. 9B). Furthermore, the effect of GO-Se exposure on morphological changes was evaluated. As shown in Fig. 9C, significant morphological abnormalities were observed in exposed embryos with increasing GO-Se concentration. Significant accumulation of GO-Se was observed at the surface of exposed embryos, particularly on the chorion. Interestingly, after 24 h of exposure, pericardial edema and swollen yolk were found at exposure concentrations of 150 μg mL^−1^ and 200 μg mL^−1^.

### 3.10 *In vivo* cellular effect of GO-Se

**Fig. 10:**
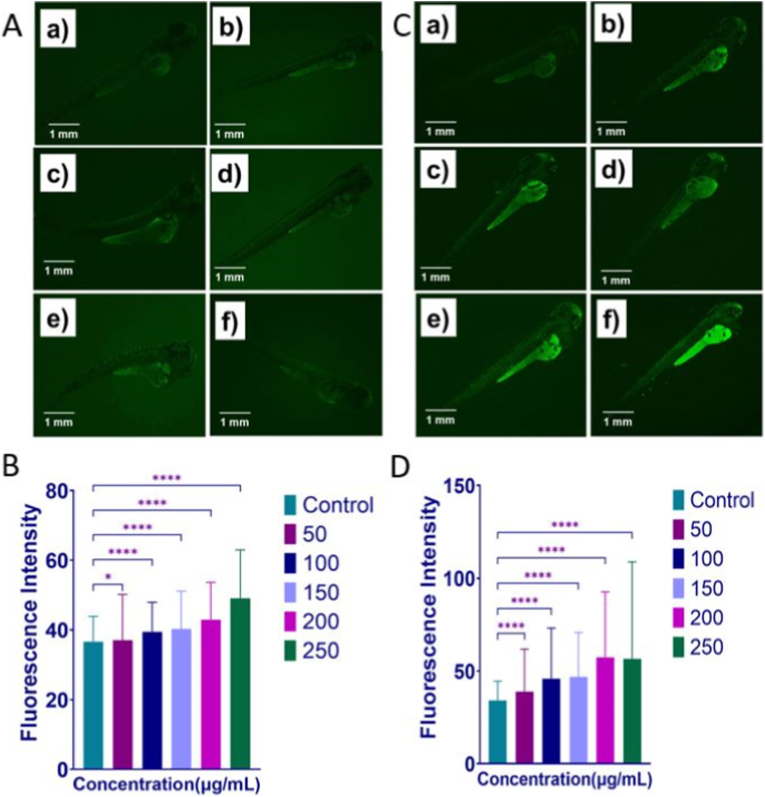
Cellular impact of GO-Se; **(A)** Fluorescence image of zebrafish embryos exposed to GO-Se for 72 h stained with H_2_DCFDA for oxidative stress evaluation. The embryos were exposed to different concentration presented as **(a)** untreated **(b)** 50 μg mL^−1^**(c)** 100 μg mL^−1^ **(d)** 150 μg mL^−1^**(e)** 200 μg mL^−1^**(f)** 250 μg mL^−1^. **(B)** Histogram presentation of quantitative fluorescence intensity of H_2_DCFDA in embryos exposed to different concentration of Go-Se. **(C)** Fluorescence image of zebrafish embryos exposed to GO-Se for 72 h stained with AO for apoptosis. The embryos were exposed to different concentration presented as **(a)** untreated **(b)** 50 μg mL^−1^**(c)** 100 μg mL^−1^ **(d)** 150 μg mL^−1^**(e)** 200 μg mL^−1^**(f)** 250 μg mL^−1^. **(D)** Histogram presentation of quantitative fluorescence intensity of AO in embryos exposed to different concentration of Go-Se. The fluorescent intensity was measured using Image J. The values are presented as mean ± SD.

The mechanistic impact of GO-Se on zebrafish embryos was evaluated by analyzing the cellular responses after zebrafish was exposed to different concentrations of GO-Se. The induced oxidative stress was first evaluated in GO-Se-exposed embryos through DCFDA staining. As shown in Fig. 10A and B, the green fluorescence of H_2_DCFDA in exposed embryos increased with GO-Se concentration, indicating dose-dependent induction of oxidative stress. Apoptosis induced by oxidative stress in GO-Se-exposed embryos was further evaluated through AO staining. As shown in Fig. 10C and D, the green fluorescence of AO significantly increased with the concentration of GO-Se, indicating more apoptotic cells in embryos.

### 3.11 DFT results

#### 3.11.1 Structural and energetic features

**Fig. 11:**
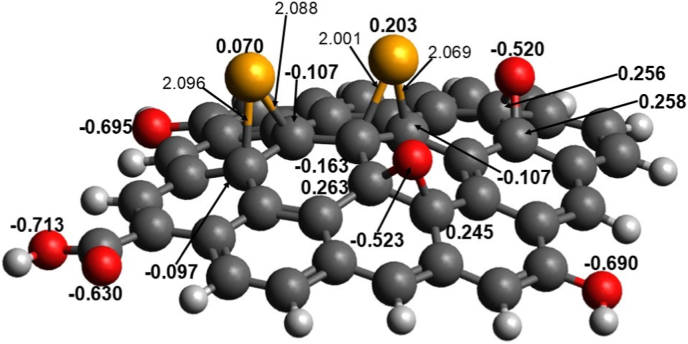
RGO-Se singlet model optimized at the B3LYP/6-31G∗ level with the implicit effects from water. Selected bond distances are given in Å. NBO charges at the selected atoms are shown in bold. Color coding: dark grey for C, light grey for H, red for O, and orange for Se. (For interpretation of the references to color in this figure legend, the reader is referred to the Web version of this article.)

Table S4 (Supplementary Data) lists the results for the RGO-Se model calculated at the B3LYP/6-31G∗ level, and Fig. 11 shows the optimized structure of the global-minimum RGO-Se singlet model. These results demonstrate that (i) the triplet structure of the RGO-Se model has a significantly higher energy than the singlet structure, with energy differences approximately 23 kcal mol^−1^ in the gas phase and 22 kcal mol^−1^ in the implicit water. (ii) The global-minimum singlet structure has noticeable HOMO/LUMO energy difference (2.17 eV in the gas phase and 2.11 eV in the implicit water). It is known that large values of the HOMO/LUMO energy differences suggest noticeable stability of compounds considered therefore, the RGO-Se system can be considered as having quite high stability in biological environments (see also below discussion of its GRPs analysis). (iii) The singlet structure has significant dipole moment (7.24 D in the gas phase and 12.09 D in the implicit water). This implies that the RGO-Se system can interact strongly with the polar moieties of various biomolecules, thus supporting the experimental results. Moreover, due to its significant dipole moment the RGO-Se system would actively interact with polar reactive oxygen species, which provides support for the experimental findings of reducing ROS levels. (iv) Upon optimization, the RGO-Se model becomes noticeably distorted with the Se = and O = groups becoming more protruding and thus potentially more accessible for interactions with solvent molecules and polar moieties of various biomolecules in the surroundings. Of course, this result should be interpreted with caution, as the C-Se bond length remains within the experimentally observed range [43].

#### 3.11.2 NBO charges, FMOs, and MEP analysis

**Fig. 12:**
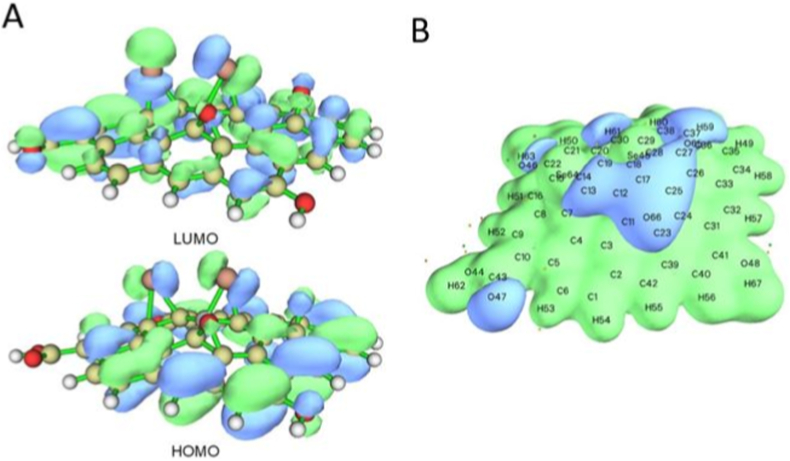
(A) FMOs and (B) MEP plot of the singlet RGO-Se structure, calculated at the B3LYP/6-31G∗ level with the implicit effects from water. Atom numbering is shown in the MEP plot. Color coding: yellowish grey for C, light grey for H, red for O, and dark orange for Se. (For interpretation of the references to color in this figure legend, the reader is referred to the Web version of this article.)

Furthermore, analysis of the NBO charges on selected atom of the models (Fig. 11) and FMOs and MEP plot (Fig. 12) shows the following features. (i) The Se-centers bear positive charges (0.070–0.203 e), whereas the C-atoms to which they are bound have negative charges (−0.097 to −0.163 e) (Fig. 11). The O-centers connected to the RGO surface as O = groups are significantly negatively charged (−0.520 to −0.523 e) with noticeable positive charges on the C-atoms to which they are bound (0.245–0.263 e). The O-centers of the OH-groups also have significant negative charges (−0.690 to −0.695 e), and the oxygens of the carbonyl group bear high negative charges as well (−0.630 to −0.713 e). These results imply that the RGO-Se system would interact strongly via electrostatic interactions and hydrogen bonding with polar moieties of biologically important molecules and with polar solvent molecules, thus supporting the experimental findings. (ii) The graphene surface, Se = groups, and O = groups contribute to the HOMO and LUMO of the RGO-Se model (Fig. 12A) and thus might be involved in various biochemical reactions. (iii) The MEP plot (Fig. 12B) of the RGO-Se model shows accumulations of positive electrostatic potential (as shown by green color) over the graphene surface, Se = groups, part of the carbonyl group, and OH-groups, as well as accumulations of negative electrostatic potential (shown by blue color) at the O = groups and O = moiety of the carbonyl group. These results suggest the electrostatic interactions between the RGO-Se system and the polar parts of biologically important models, as well as with the polar solvent molecules, thus supporting the experimental findings.

#### 3.11.3 QTAIM analysis

**Fig. 13:**
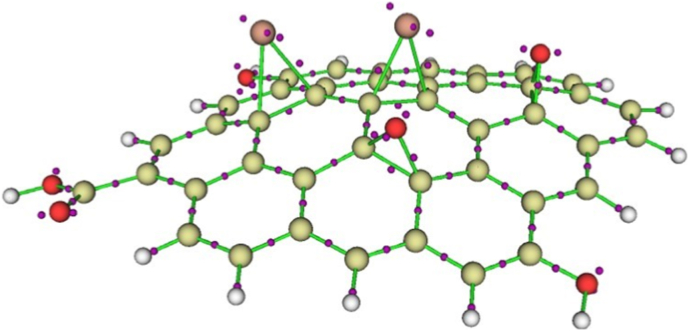
QTAIM analysis results for the RGO-Se model calculated at the B3LYP/6-31G∗ level with the implicit effects from water. Violet dots designate electron pairs, either bonding or lone. (For interpretation of the references to color in this figure legend, the reader is referred to the Web version of this article.)

QTAIM analysis results demonstrated in Fig. 13 show the presence of lone pairs on the Se- and O-atoms which can participate in electrostatic interactions and hydrogen bonding with polar solvent molecules and polar moieties of biomolecules in the environment, as well as play a role in the chemical reactions of the RGO-Se system.

#### 3.11.4 GRPs analysis

The ionization potential *(IP)*, electron affinity *(EA)*, global hardness (*η),* global electronegativity (*X*), global softness (*σ*), global electrophilicity (*ω*), propensity to donate electron (*ω*-), and propensity to accept electron (*ω+*) were computed using the FMOs energies according to Equations (Supplementary Data, Equations), and their computed values in *eV* are presented in Table 1.

**Table 1:** GRPs for the RGO-Se model (*eV*) calculated at B3LYP/6-31G∗ level with the implicit effects from water.

IP	EA	Gap	X	η	μ	σ	ω	ω+	ω-
2.93	5.04	2.11	3.989	2.111	−3.989	0.474	3.769	2.038	6.028

Analysis of the GRP values given in Table 1 shows the following features. (i) The RGO-Se system has a noticeable *IP* value (2.93 eV) and much more pronounced *EA* value (5.04 eV), which implies that it should act as a good electron acceptor in reactions with biologically important molecules. (ii) The noticeable HOMO/LUMO *Gap* value (2.11 eV) and *μ* value (−3.989 eV) imply the RGO-Se system as having quite high stability in biological environments. (iii) Furthermore, *η* value and *σ* value of the system are moderate (2.111 eV and 0.474 eV, respectively), which implies that the RGO-Se system might not be very reactive in biological environments, depending on the reaction conditions. (iv) Next, *X* and *ω* values are significant (3.989 eV and 3.769 eV, respectively), which along with the high *ω-* (6.028 eV), compared to the value of *ω+* (2.038 eV), and the high *EA* value, (5.04 eV) imply that the RGO-Se system in redox processes with biologically important molecules would act as a pronounced acceptor of electrons. Thus, it can interact with free radicals, inducing various cellular damage, including DNA damage. Moreover, due to its increased electrophilicity, the RGO-Se system would actively interact with radical reactive oxygen species, which provides support for the experimental findings of reducing ROS levels. Therefore, the GRP analysis results provide support for the experimental data as well.

## 4 Discussion

Our findings demonstrated that GO-Se exhibited a dose- and time-dependent cytotoxic effect in NHDF cells. The progressive decline in cell viability over time indicated an increasing susceptibility of NHDF cells to GO-Se exposure, a trend consistent with previous reports on the dose-dependent toxicity of nanomaterials in fibroblast cells [44]. Wang et al. (2010) demonstrated that GBNs at concentrations exceeding 50 μg mL^−1^ caused a significant reduction in fibroblast viability over 1–5 days, primarily through apoptotic induction and oxidative stress [44], which aligns with our observations of GO-Se. Se-functionalization appears to modulate oxidative stress responses, contributing to its characteristic dose-dependent effects. Specifically, GO-Se reduced ROS levels by accelerating peroxide decomposition under conditions of high oxidative stress, while paradoxically increasing ROS levels when the basal ROS concentration was low [16]. This dual redox behavior suggests that GO-Se can serve as both an antioxidant and a pro-oxidant, depending on the cellular microenvironment and exposure levels. Similarly, Tuyen et al. (2023) and Kakhki et al. (2024) reported that Se-functionalized GO exhibited protective effects at moderate doses but induced cytotoxicity at higher concentrations, particularly in cancer cells [45,46]. This aligns with our results, where NHDF cells tolerated GO-Se at intermediate doses but exhibited significant viability reduction at higher concentrations. Interestingly, GO-Se treatment led to a significant reduction in intracellular ROS levels, as shown by H_2_DCFDA staining and flow cytometry analysis. Huang et al. (2017) demonstrated that GO-Se exhibited a strong GPx-like catalytic efficiency, effectively neutralizing H_2_O_2_ and providing cytoprotection against oxidative stress in RAW264.7 cells, with no significant toxicity observed at the tested concentrations. Se-functionalization appears to play a key role in balancing oxidative stress, acting as an antioxidant under high ROS conditions and promoting ROS generation in low ROS environments [18]. Additionally, Nam et al. (2024) demonstrated that phytocompound-mediated GO-Se synthesis enhanced antioxidant and anti-inflammatory properties, which could explain the protective effects of GO-Se in normal cells [15]. Se nanoparticles are believed to participate in complex reactions to assimilate and form selenoproteins, which can modify the antioxidant activity and serve as enhancers for the immune response toward the inflammatory action [47]. Interestingly, despite the cytotoxicity of GO-Se at IC_50_, we observed the formation of compact spheroid-like structures in NHDF cells at 24 h, 72 h, and 168 h (Supplementary Data, Fig. S3). These spheroids were smaller than those in control groups, suggesting impaired proliferation and possible stress-induced aggregation. Such morphological alternations may reflect an adaptive survival mechanism involving altered adhesion and extracellular matrix (ECM) remodeling, under GO-Se-induced redox stress. Similar phenomena have been reported in fibroblast-based 3D models exposed to oxidative or chemical stressors [48,49]. The less compact structure supports the notion of a stress-adaptive response, in which oxidative stress and cytokine modulation alter cell–cell and cell–matrix interactions. Collectively, these findings indicate that GO-Se not only affects fibroblast viability but also compromises their ability to organize into 3D multicellular structures, highlighting the importance of cell type and redox context in determining cellular responses and underscoring the need for further mechanistic studies. Building on these observations, we found that GO-Se treatment delayed fibroblast migration compared with controls, as shown by a sustained reduction in wound closure over time. This impairment of fibroblast motility likely reflects cytoskeletal alterations and stress responses, consistent with reduced cell viability and further supports the cytotoxic profile observed in our study. Previous reports also described that GO exposure disrupts actin filament organization and reduces epithelial cell migration due to cytoskeletal remodeling [50,51] supporting our interpretation. Therefore, the migration assay should be interpreted as a functional endpoint of biocompatibility, rather than as evidence of wound healing properties.

GO has been extensively studied for its ability to generate ROS, which can lead to oxidative stress-related cytotoxicity [52,53]. However, Se-functionalization appears to modulate GO redox properties, influencing its impact on cellular ROS balance. Our results revealed that GO-Se treatment led to a dose- and time-dependent suppression of ROS levels in NHDF cells, as shown by flow cytometry and fluorescence microscopy analyses. A significant reduction in ROS accumulation was observed at higher GO-Se concentrations and longer exposure times. Xia et al. (2017) reported that GO-Se exhibited a dual function in ROS regulation, acting as an antioxidant at high ROS levels while enhancing ROS production at low ROS levels [16]. This phenomenon aligns with our findings, where GO-Se reduced ROS levels in NHDF cells, possibly by facilitating peroxide decomposition. Similarly, Huang et al. (2017) demonstrated that GO-Se exhibited GPx-like catalytic activity, effectively neutralizing H_2_O_2_ and providing cytoprotection against oxidative stress [18]. These findings suggest that the redox behavior of GO-Se depends on the cellular oxidative state, potentially mitigating oxidative stress-induced cytotoxicity at specific concentrations. Furthermore, Kazemian Kakhki et al. (2024) reported that the ROS modulation effect of Se-functionalized GO depended on the cell type and microenvironment. They demonstrated that β-cyclodextrin-GO loaded with Se significantly increased ROS levels in U87 glioblastoma cells, which led to oxidative stress and apoptosis [46]. Therefore, the GO-Se impact on ROS dynamics is highly cell-type-dependent, exhibiting antioxidant effects in fibroblasts while promoting oxidative stress in cancerous cells. These findings suggest that GO-Se does not universally act as an ROS suppressor or inducer but rather modulates ROS based on cellular conditions. Additionally, a dose-dependent decrease in CAT and GPx activities was observed in NHDF cells at all examined time points following GO-Se treatment. This response contrasts with that of unmodified GO, which typically induces oxidative stress and upregulates antioxidant defenses, including CAT activity [54,55]. The reduction in enzymatic activity observed after GO-Se exposure is likely attributed to its GPx-mimicking catalytic properties, which enable ROS detoxification independently of the cellular enzymatic systems, as reported for Se-functionalized materials [18,56]. Similar suppressive effects on endogenous antioxidant enzymes have also been documented under prolonged exposure to antioxidant-rich compounds, suggesting a feedback downregulation of enzymatic activity under low-ROS conditions [25]. These findings indicate that Se-functionalization not only modifies GO's redox reactivity but also actively reprograms the cellular antioxidant response.

GO has been shown to induce DNA damage through multiple mechanisms, including ROS generation and direct interaction with DNA, leading to genotoxic stress in various cell types [[57], [58], [59]]. Previous studies have also demonstrated that GBNs can modulate DNA repair and checkpoint control pathways [60,61]. In our study, GO-Se exposure altered the expression of DDR proteins in NHDF cells, indicating activation of repair responses but also disruption of checkpoint control. Upregulation of repair-associated proteins such as Human apurinic/apyrimidinic endonuclease (*APE1*), Serine/Threonine kinase (*ATR*), BReast CAncer gene 2 (*BRCA2*), and DNA-dependent protein kinase catalytic subunit (*DNA-PKcs*), Poly (ADP-ribose) polymerase (*PARP*) points to compensatory mechanisms aimed at maintaining genomic stability. Strobel et al. (2017) described that *APE1* promotes HR fidelity while limiting error-prone non-homologous end joining (NHEJ) [60], and Shrivastav et al. (2008) reviewed the central role of *DNA-PKcs* and *BRCA2* in coordinating HR and NHEJ [62]. The concurrent downregulation of Checkpoint kinase1(*Chk1*) together with increased Checkpoint kinase 2 (*Chk2*), G2/mitotic-specific (*Cyclin B1*), and Serine/threonine-protein kinase (*PLK1*) suggests altered checkpoint regulation and continued cell cycle progression despite DNA damage, in line with reports that GBNs can perturb cell cycle regulators [61]. Previous studies also showed that GO exposure increases *γ-H2AX*, *ATR*, and *p53* signaling [59,63], and that it activates TP53 and apoptosis in fibroblasts [64]. In our data, the induction of MutS Homolog 2 (*MSH2*) and Nibrin (*Nbs1*) further highlights enhanced lesion recognition, while Optineurin (*OPTN*) upregulation may suggest cross-talk between DDR and autophagy. Se-functionalization may additionally influence DDR as Se compounds can activate Ataxia-telangiectasia mutated (ATM)-dependent DDR pathways, particularly through mismatch repair protein MutL homolog 1 (*hMLH1*), which may contribute to the enhanced DNA repair efficiency [65]. Taken together, these results indicate that GO-Se triggers genotoxic stress accompanied by both activation of DNA repair proteins and partial disruption of checkpoint signaling. Although Se incorporation may provide protective effects, the long-term implications for genomic stability remain uncertain and warrant further investigation.

GO and Se have been widely investigated for their immunomodulatory properties. The immunological effects of GO are strongly dependent on its physicochemical characteristics, concentration, and cellular context, with studies demonstrating its ability to exert both pro-inflammatory and immunosuppressive effects [[66], [67], [68]]. GO influences immune responses primarily through its interaction with antigen-presenting cells, leading to T-cell activation and cytokine modulation [69]. Meanwhile, Se plays a key role in immune regulation, primarily through its incorporation into selenoproteins, which modulate oxidative stress and inflammatory pathways [70]. Se supplementation has been shown to reduce pro-inflammatory cytokines and mitigate oxidative stress-induced inflammation [[71], [72], [73]]. These anti-inflammatory effects are largely attributed to the inhibition of Nuclear Factor kappa-light-chain-enhancer of activated B cells (NF-κB) and the antioxidant activity of selenoproteins, particularly glutathione peroxidase, which regulates inflammation and immune signaling [70,74]. Given these properties, GO-Se is expected to enhance both immunoregulatory and antioxidant mechanisms, leading to a controlled cytokine response. Nam et al. (2024) demonstrated that GO-Se exhibits strong anti-inflammatory activity, likely by preventing protein denaturation and oxidative stress-induced cellular damage [15]. Their findings align with previous research indicating that Se-functionalization enhances the bioactivity and stability of GBNs, highlighting their potential for immune modulation [16,75].

In this study, GO-Se significantly altered cytokine expression, leading to the upregulation of key chemokines (C-C motif chemokine 4 (*MIP-1β), Eotaxin,* and C-C motif chemokine 1 (*I-309*), interleukin (IL)s (*IL-1α, IL-1ra, IL-2, IL-5, IL-15,* and *IL-16*), and the matrix metalloproteinase inhibitor (*TIMP-1*). This cytokine profile suggests enhanced T-cell activation and chemokine-mediated immune signaling. *IL-2* and *IL-15* are essential for T-cell proliferation and survival, playing critical roles in effector and memory T-cell homeostasis [76]. *IL-5* is associated with T-helper-2 (Th2) differentiation and immune homeostasis, influencing eosinophil activation [77], while *IL-1α* enhances T-cell activation and antigen-presenting cell function, linking innate and adaptive immune responses [78]. The upregulation of *IL-16* and *MIP-1β* further supports T-cell recruitment and migration, reinforcing the role of GO-Se in adaptive immune regulation [79]. Additionally, *TIMP-1* upregulation suggests a role in T-cell survival and extracellular matrix remodeling, which may impact tissue repair and immune homeostasis [80]. At the same time, several cytokines were downregulated. The observed reduction in Tumor necrosis factor alpha (*TNF-α*) and *IL-8* levels suggests that the NF-κB inhibitory effects of Se suppress excessive inflammation while preserving adaptive immune responses [70]. This dual regulatory effect aligns with reports demonstrating that Se modulates pro-inflammatory cytokine release and oxidative stress responses [71,73].

Modifications of GO surface properties have been shown to alter its immunogenicity. Polyvinylpyrrolidone-coated GO has been reported to reduce dendritic cell activation, decreasing *TNF-α* and *IL-1β* levels while maintaining *IL-6* secretion [81]. These findings suggest that Se-functionalization may similarly modulate GO immunological interactions, potentially shifting the balance between pro-inflammatory and immunosuppressive responses. Additionally, GO-antisense miRNA-21 complexes have been shown to alter cytokine expression in glioblastoma models, reducing intercellular adhesion molecule 1 (*ICAM-1)* levels and modulating *IL-6* and *IL-8* secretion, further underscoring the capacity of GO-based materials to regulate immune signaling across different biological contexts [82]. GO-Se may also interact with Toll-like receptor (TLR) pathways, modulating inflammatory cytokine expression and downstream immune signaling. Prior studies indicate that TLR activation is a key driver of GO-induced immune responses [83]. Dudek et al. reported that GO stimulates TLR signaling, leading to *IL-1α*, *IL-6*, *IL-10*, and *TNF* secretion in macrophages [84]. Consistently, we observed upregulation of *IL-1α* and *IL-1ra* following GO-Se treatment, suggesting that GO-Se engages TLR pathways similarly to GO. Furthermore, Cebadero-Dominguez et al. (2023) demonstrated that rGO upregulated *IL-6* while suppressing *TNF-α* in monocytes, a trend that aligns with our findings [85]. Taken together, these findings highlight that GO-Se exhibits a dual immunomodulatory effect, enhancing adaptive immunity while attenuating acute inflammatory responses. Se-functionalization introduces antioxidant and NF-κB inhibitory properties, refining immune regulation. By modulating cytokine expression, T-cell activation, and TLR signaling, GO-Se emerges as a promising candidate for biomedical applications for immune modulation.

The evaluation of GO-Se toxicity in zebrafish embryos provides critical insights into its biocompatibility. Our findings indicate a concentration-dependent decline in survivability, suggesting that the exposure to GO-Se at concentrations ≥100 μg mL^−1^ compromises embryonic viability. Moreover, the time-dependent survivability of the embryos on GO-Se exposure was also observed. This observation aligns with previous studies reporting GO-induced developmental toxicity through mechanisms involving oxidative stress, DNA modification, and protein carbonylation at environmentally relevant concentrations [9,86].

Heart rate analysis revealed a significant decrease in cardiac function at concentrations ≥100 μg mL^−1^, indicating potential cardiotoxic effects of GO-Se. This is consistent with previous studies demonstrating GO-induced pericardial edema, arrhythmia, and oxidative stress-mediated cardiac dysfunction in zebrafish embryos [9,87]. Mechanistically, GO has been shown to cause mitochondrial dysfunction and excessive ROS generation, leading to cardiovascular toxicity [7,9]. Similarly, GO-Se appears to disrupt heart development, likely through the induction of mitochondrial oxidative stress and lipid peroxidation, though Se's antioxidant properties might partially mitigate these effects.

Morphological abnormalities such as pericardial edema, and body curvature were also observed, with severity increasing at higher concentrations. The abnormalities can be attributed to the accumulation GO-Se on the surface of chorion leading to blockage of the chorion pores which can be further reasoned for the abnormal oxidative stress condition due to hypoxic condition inside embryos. These findings suggest that GO-Se interferes with key developmental pathways, leading to teratogenic effects. Previous studies on GO have documented similar effects, including craniofacial malformations, eye defects, and tail flexure [88]. Additionally, Se-containing compounds like selenomethionine have been reported to induce developmental toxicity in zebrafish embryos, primarily through oxidative stress and the unfolded protein response [89].

Oxidative stress plays a central role in the toxicity observed in GO and its derivatives. Our fluorescence imaging with H_2_DCFDA staining confirmed a dose-dependent increase in ROS production, aligning with studies that demonstrate oxidative damage following GO exposure [86,90]. GO has been reported to induce oxidative stress due to elicited hypoxic condition because of the chorion blockage. GO nanoparticles penetrate the chorion of zebrafish embryos, leading to hypoxia, delayed hatching, and developmental toxicity mediated by oxidative stress [88]. Furthermore, excessive ROS generation from GO exposure leads to lipid peroxidation, DNA damage, and apoptotic cell death [9,86]. GO nanoparticles can also accumulate in various zebrafish organs, affecting their development and behavior [86]. Interestingly, the oxidative stress effect was found to be affected in contrasting manner compared to the *in vitro* results. The discrepancy can be attributed to the dual redox activity of Se. Se-containing nanomaterials act as antioxidants under elevated oxidative stress but may exert pro-oxidant effects in reductive or hypoxic environments. In the zebrafish model, GO-Se accumulation on the chorion surface may have restricted oxygen diffusion, leading to local hypoxia and secondary ROS overproduction, a mechanism previously proposed for GO-induced embryotoxicity [7,91]. Together, these observations emphasize that the redox-modulating effects of GO-Se are highly context dependent, varying with both the biological system and exposure conditions.

Apoptotic analysis confirmed GO-Se-induced apoptosis in zebrafish embryos, particularly at concentrations between 150 and 250 μg mL^−1^. The presence of apoptotic cells suggests the activation of intrinsic apoptotic pathways, potentially through mitochondrial disruption and caspase activation. GBNs have been previously shown to induce oxidative stress, DNA damage, and inflammation, all of which are involved in the mitochondria-mediated apoptosis pathway [92,93].

The DFT calculations provided complementary evidence to support the experimental observations. The singlet RGO-Se structure was found to be energetically more stable than the triplet form, with a HOMO–LUMO *Gap* of ∼2.1 eV, suggesting a thermodynamically stable but still reactive system [94]. Importantly, the calculated high dipole moment values (7–12 D) indicate strong polarity of the structure, which would facilitate interactions with important biomolecules such as DNA, proteins, and phospholipids [95].

Furthermore, the GRPs demonstrated that GO-Se would act as a pronounced electron acceptor, consistent with its ability to scavenge or redirect ROS [94]. This dual redox capacity may explain why GO-Se decreased ROS in fibroblasts but increased ROS in zebrafish embryos, depending on the redox environment [96]. The presence of negatively charged oxygen groups and polarized Se-centers further supports potential interactions with DNA and other cellular targets, in line with the observed genotoxic responses [97].

Overall, the computational results strengthen the biological findings by providing a molecular-level rationale: GO-Se is a stable yet highly polar and electron-accepting system, properties that underline its context-dependent antioxidant/pro-oxidant activity and its ability to interact with cellular macromolecules.

### 4.1 Limitation

The present study has several limitations. First, pristine GO and Se-only controls (e.g., sodium selenite or SeNPs) were not included, which restricts the ability to fully disentangle the respective contributions of GO and Se. Second, the *in vivo* assessments were restricted to morphological, ROS, apoptotic, and cardiac endpoints, without molecular markers such as qPCR (quantitative polymerase chain reaction)-based gene expression analysis. Third, our findings are based primarily on *in vitro* fibroblast and *in vivo* zebrafish embryo models, which may not fully capture the complexity of mammalian physiological and systemic responses. Furthermore, the concentration ranges applied, while suitable for defining IC_50_ values and mechanistic endpoints, may not directly correspond to environmentally or clinically relevant exposures. Finally, the dual redox behavior of GO-Se complicates extrapolation of results, emphasizing the need for long-term studies with integrative multi-omics approaches to clarify systemic outcomes and translational potential.

## 5 Conclusion

In conclusion, this study demonstrates that GO-Se combines specific electronic reactivity, predicted by DFT modeling, with distinct biological effects. In fibroblasts, GO-Se induced dose- and time-dependent cytotoxicity, impaired migration, disrupted DDR signaling, and triggered apoptosis via oxidative stress. Importantly, it suppressed intracellular ROS in fibroblasts but increased ROS generation in zebrafish embryos, reflecting its dual redox behavior and dependence on the cellular microenvironment. In addition, GO-Se modulated cytokine expression, revealing that its effects extend beyond redox regulation to include immune and inflammatory responses. Zebrafish assays further showed concentration-dependent developmental toxicity, supporting the context-dependent nature of its biological impact. Collectively, these findings indicate that GO-Se possesses specific biological properties, with implications for cytotoxicity, redox modulation, and immune responses.

## CRediT authorship contribution statement

**Tuba Oz:** Writing – review & editing, Writing – original draft, Validation, Methodology, Investigation, Conceptualization. **Suresh K. Verma:** Writing – review & editing, Writing – original draft, Methodology, Investigation, Conceptualization. **Aleksey Kuznetsov:** Writing – review & editing, Writing – original draft, Methodology. **Palaniappan Nagarajan:** Writing – review & editing, Writing – original draft, Methodology, Investigation. **Ivan Cole:** Writing – review & editing, Methodology. **Shaikh Sheeran Naser:** Writing – review & editing, Writing – original draft, Visualization, Methodology. **Krzysztof Książek:** Writing – review & editing, Methodology. **Hong Yin:** Writing – review & editing. **Małgorzata Kujawska:** Writing – review & editing, Writing – original draft, Supervision, Project administration, Methodology, Conceptualization.

## Declaration of competing interest

The authors declare that they have no known competing financial interests or personal relationships that could have appeared to influence the work reported in this paper.

## Data Availability

Data will be made available on request.
