# Supplementary material for: Selenium-modified graphene oxide: A tri-dimensional study of its cytotoxicity and developmental effects
Source: Mater Today Bio. 2025 Dec 8;36:102650. doi: 10.1016/j.mtbio.2025.102650 (PMC12756035; doi:10.1016/j.mtbio.2025.102650)
Supplement: Multimedia component 1 [file mmc1.docx]

**Supplementary Information for**

**Selenium-Modified Graphene Oxide: A Tri-Dimensional Study of Its Cytotoxicity and Developmental Effects**

Tuba Oz^a,b^*, Suresh K. Verma^c^, Aleksey Kuznetsov^d^, Palaniappan Nagarajan^e^, Ivan Cole^f^, Shaikh Sheeran Naser^c^, Krzysztof Książek^g^, Hong Yin^f^, Małgorzata Kujawska^a^*

**Figure S1:** The Particle Size Distribution of GO-Se


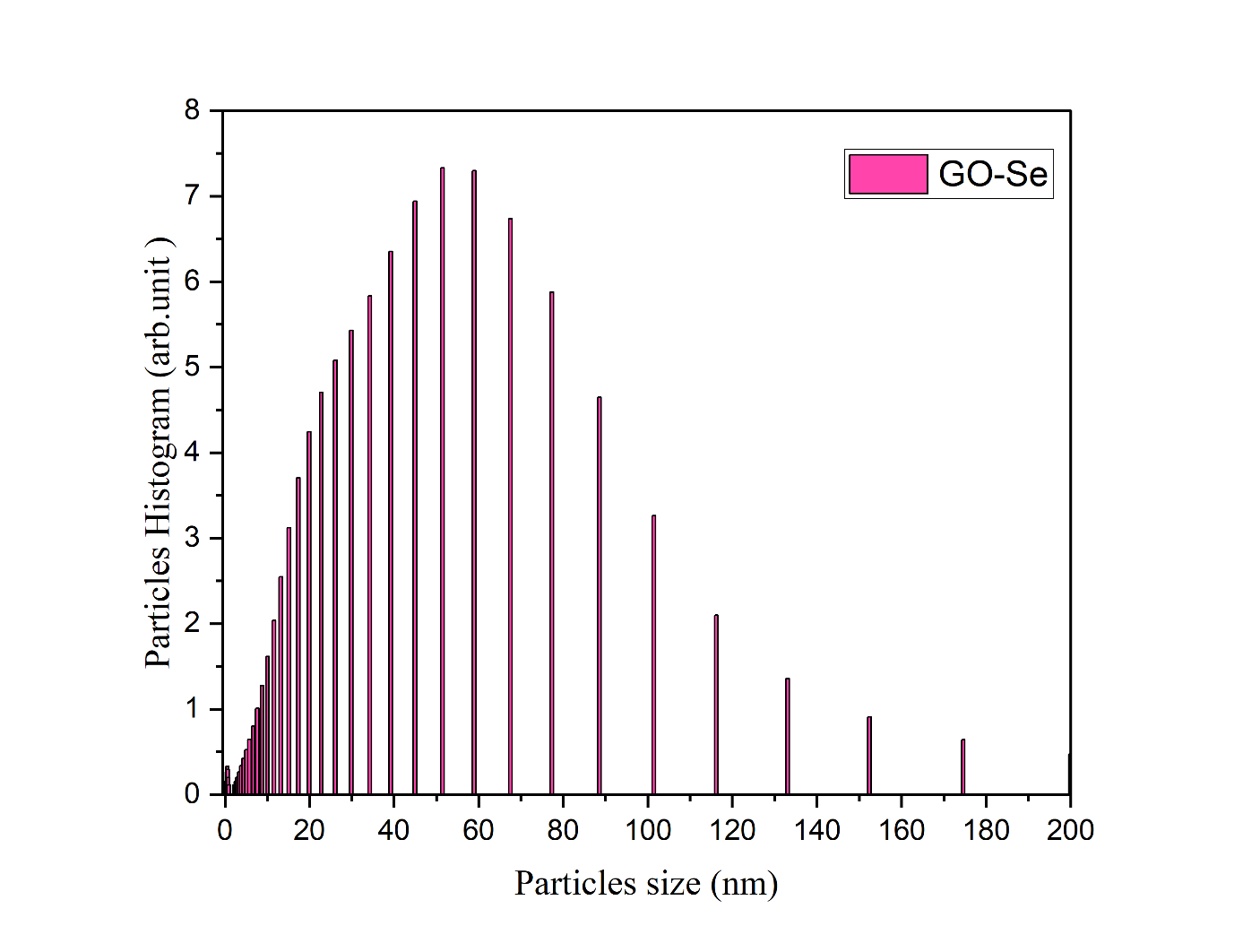


**Figure S1.** The Dynamic light scattering (DLS) size distribution histogram reveals that GO-Se exhibits a predominant nanoparticle population within the size range of approximately 50–70 nm, indicating that the majority of particles are well within the nanoscale.

**Figure S2:** Effect of GO-Se on Cell Morphology


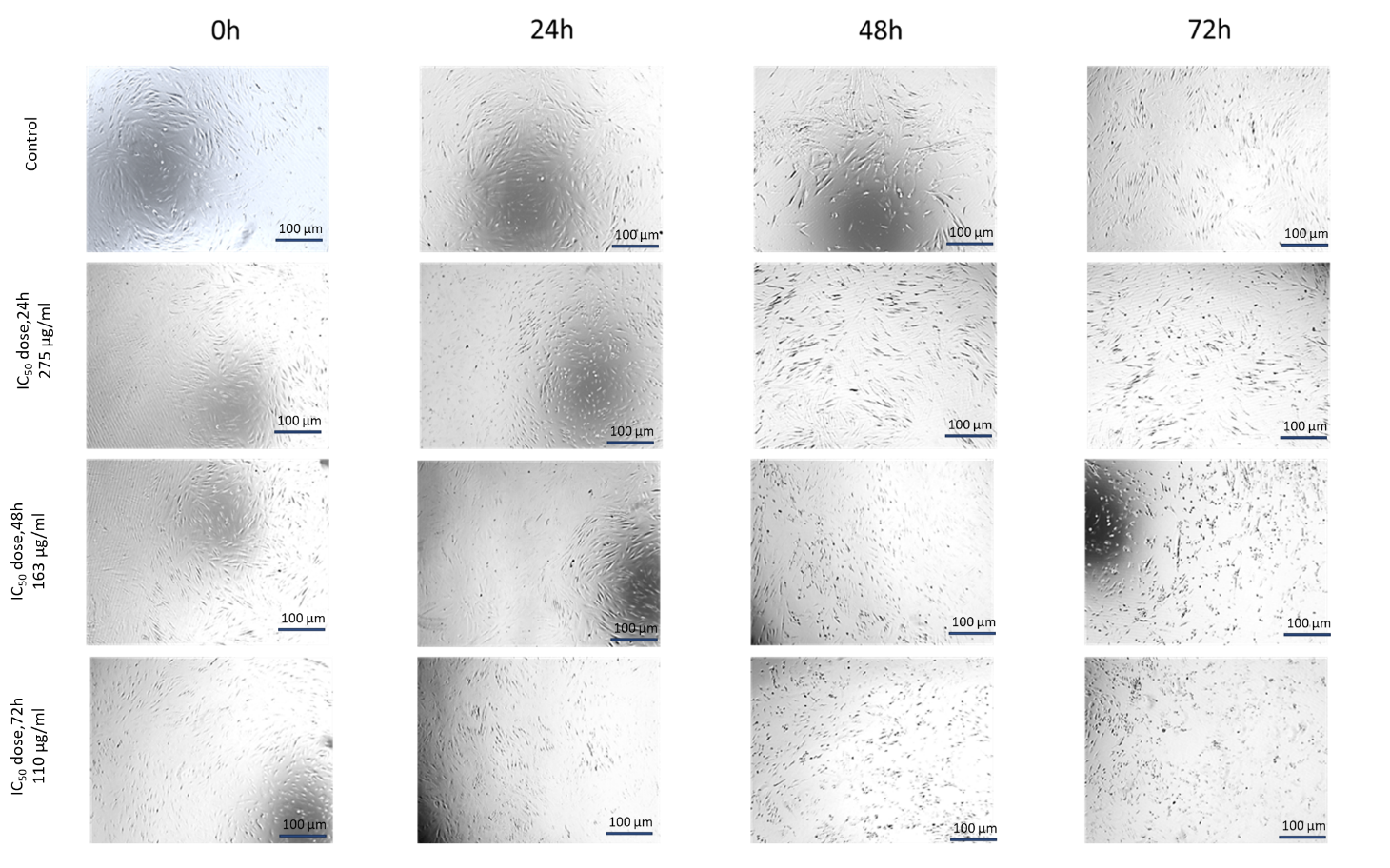


**Figure S2.** Morphological observation by inverted microscope (×10). NHDF cells were seeded in 6-well plates (2.5×10⁵ cells/well) and incubated at 37°C with 5% CO₂ for 24 h. Cells were then treated with IC_50_ doses of GO-Se for 24 h, 48 h, and 72 h. Morphological changes were observed using an inverted microscope (Nikon Eclipse TS100, USA).The scale bar is 100μm.

**Figure S3:** Spheroid Growth and Morphology

**A.**

**
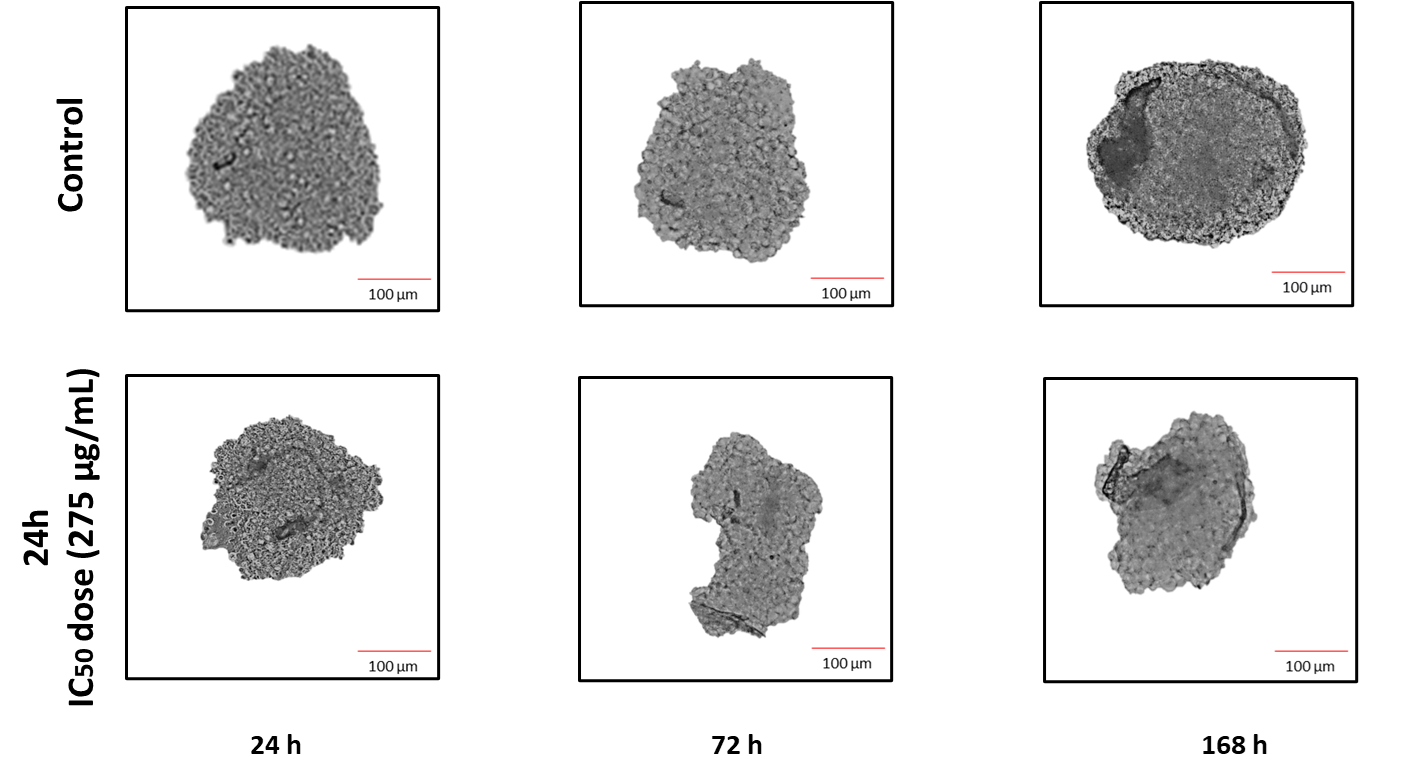
**

**B.**
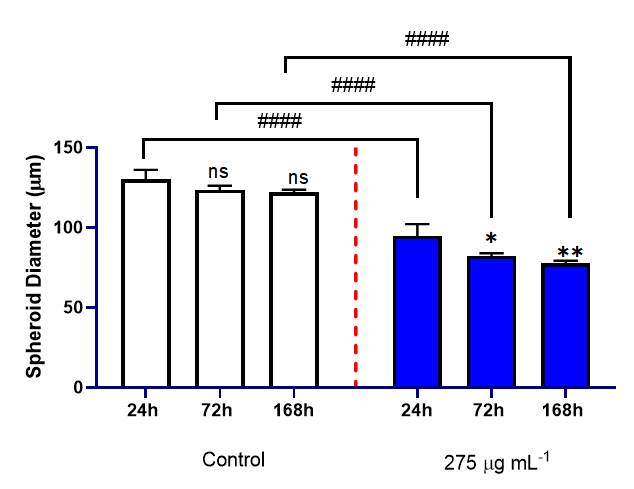


**Figure S3. (A)** Spheroid growth during NHDF cell culture observed at 24 h, 72 h, and 168 h. Scale bar: 100 μm. Magnification ×40. Morphological changes of spheroid were observed using an inverted microscope (Nikon Eclipse TS100, USA). **(B)** The formed spheroids were of different sizes; their diameters were between 70 and 140 μm. Data are reported as means of three independent experiments ±SD by one-way ANOVA test with Tukey’s comparison test. ns: not significant, *p<0.05, **p<0.01 vs 24 h; #### p<0.0001(Control vs 275 μg mL^-1^).

**Figure S4:** Survivability rate of embryonic zebrafish exposed to different concentrations of GO-Se


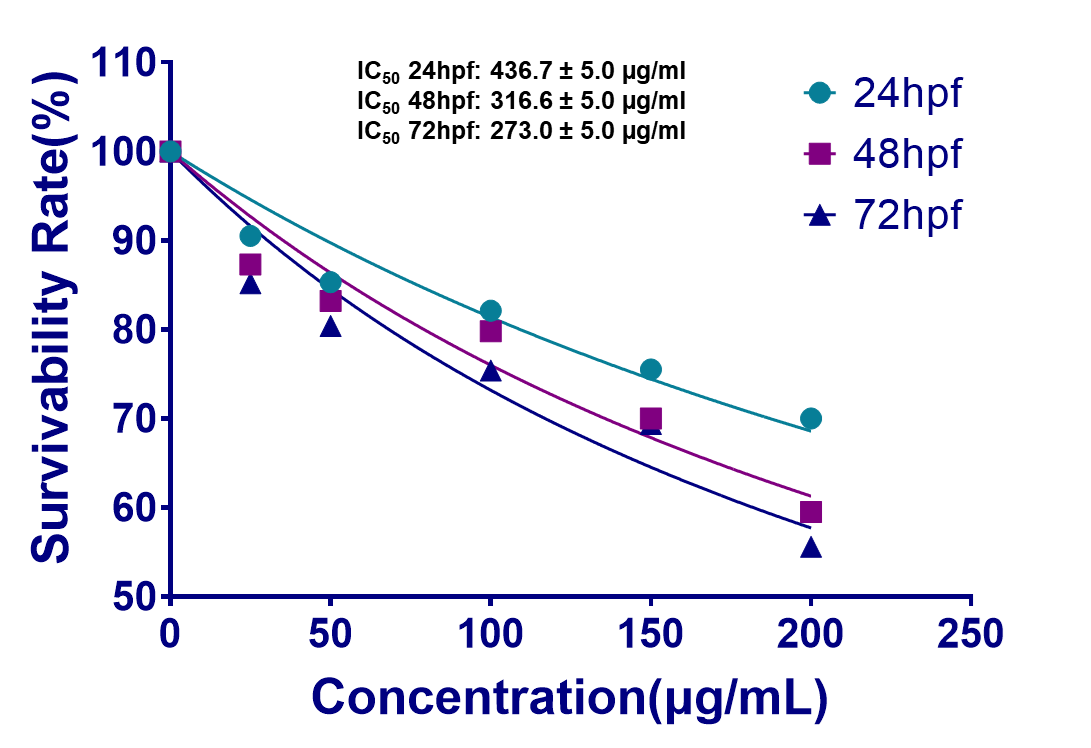


**Figure S4.** *In vivo* physiological toxicological effect of GO-Se on embryonic zebrafish. The IC_50_ value of GO-Se was calculated as 436.7 ± 5.0 µg mL^-1^, 316.6 ± 5.0 µg mL^-1^, and 273.0 ± 5.0 µg mL^-1^ for 24 h post-fertilization (hpf), 48 hpf, and 72 hpf embryos.

**Table S1: Characterization of GO-Se Composites**

| **Property** | **Method/Source** | **Results** | **Remarks** |
| --- | --- | --- | --- |
| Size (thickness) | TEM | 50–80 nm (GO sheet thickness) | Attributed to ultrasonication-induced exfoliation and porosity formation |
| Distribution | FESEM + EDX Mapping | Uniform distribution of Se nanorods on GO sheets | Electrostatic and hydrogen bonding interactions facilitate even decoration |
| Zeta Potential / Stability | Not directly reported; inferred from electrochemical studies | Stability supported by strong Se–GO interactions | Electrochemical OCP, impedance, and polarization indicate good surface stability |
| Surface Morphology | FESEM & TEM | Se nanorods anchored to porous GO surfaces | Interactions with epoxy, hydroxyl, and carboxyl groups confirmed by Raman/FTIR; coatings showed smooth, pit-free alloy surfaces after immersion tests |

*Field Emission Scanning Electron Microscopy (FESEM), Transmission Electron Microscopy (TEM), Fourier-Transform İnfrared Spectroscopy (FTIR), Energy-Dispersive X-Ray Spectroscopy (EDX), Open Circuit Potential (OCP)*

**Table S2**. Human DNA Damage Response Antibody Array

| **Target protein** | **Target protein name** |
| --- | --- |
| POS | Positive Control Spot |
| NEG | Negative Control Spot |
| *APE 1* | Human apurinic/apyrimidinic endonuclease |
| *ATR* | Serine/Threonine Kinase |
| *BRCA1* | BReast CAncer gene 1 |
| *BRCA2* | BReast CAncer gene 2 |
| *c-Abl* | Abelson tyrosine kinase |
| *CDC25A* | Cell division cycle 25A |
| *CDK1* | Cyclin-dependent kinase 1 |
| *CDK7* | Cyclin-dependent kinase 7 |
| *Chk1* | Checkpoint kinase1 |
| *Chk2* | Checkpoint kinase 2 |
| *Cyclin-B1* | G2/mitotic-specific |
| *DNA-PKcs* | DNA-dependent protein kinase catalytic subunit |
| *ERCC1* | DNA excision repair protein |
| *GADD153* | Growth arrest and DNA damage 153 |
| *Ku70* | ATP-dependent DNA helicase 2 subunit 1 |
| *Ku80* | ATP-dependent DNA helicase II subunit 2 |
| *MDM2* | Mouse double minute 2 |
| *MGMT* | Methylated DNA protein cysteine methyltransferase |
| *MSH2* | MutS Homolog 2 |
| *Nbs1* | Nibrin |
| *OPTN* | Optineurin |
| *p21* | CDKN1A cyclin dependent kinase inhibitor 1A |
| *p53* | Tumor protein P53 |
| *PARP* | Poly (ADP-ribose) polymerase |
| *PLK1* | Serine/threonine-protein kinase |
| *PPM1D* | Protein phosphatase 1D |
| *Rad17* | Cell cycle checkpoint protein |

**Table S3.** Human Inflammation Array

| **Target Protein** | **Target Protein Name** |
| --- | --- |
| *POS1, POS2* | Positive Control Spot1 and Spot2 |
| *BLC* | CXCL13, C-X-C motif chemokine 13 |
| *Eotaxin1* | CCL11, Eotaxin |
| *Eotaxin2* | CCL24, C-C motif chemokine 24 |
| *G-CSF* | CSF3, Granulocyte colony-stimulating factor |
| *GM-CSF* | CSF2, Granulocyte-macrophage colony-stimulating factor |
| *I-309* | CCL1, C-C motif chemokine 1 |
| *ICAM-1* | Intercellular adhesion molecule 1 |
| *IFNg* | IFNG, Interferon gamma |
| *IL-1a* | IL1A, Interleukin-1 alpha |
| *IL-1b* | IL1B, Interleukin-1 beta |
| *IL-1ra* | IL1RN, Interleukin-1 receptor antagonist protein |
| *IL-2* | Interleukin-2 |
| *IL-4* | Interleukin-4 |
| *IL-5* | Interleukin-5 |
| *IL-6* | Interleukin-6 |
| *IL-6sR* | Interleukin-6 receptor subunit alpha |
| *IL-7* | Interleukin-7 |
| *IL-8* | Interleukin-8 |
| *IL-10* | Interleukin-10 |
| *IL-11* | Interleukin-11 |
| *IL-12p70* | Interleukin-12 subunit alpha |
| *IL-12p40* | Interleukin-12 subunit beta |
| *IL-13* | Interleukin-13 |
| *IL-15* | Interleukin-15 |
| *IL-16* | Interleukin-16 |
| *IL-17* | Interleukin-17 |
| *MCP-1* | CCL2, C-C motif chemokine 2 |
| *MCSF* | Macrophage colony-stimulating factor 1 |
| *MIG* | C-X-C motif chemokine 9 |
| *MIP-1a* | CCL3, C-C motif chemokine 3 |
| *MIP-1b* | CCL4, C-C motif chemokine 4 |
| *MIP-1d* | CCL15, C-C motif chemokine 15 |
| *PDGF-BB* | Platelet-derived growth factor subunit B |
| *RANTES* | CCL5, C-C motif chemokine 5 |
| *TIMP-1* | Metalloproteinase inhibitor 1 |
| *TIMP-2* | Metalloproteinase inhibitor 2 |
| *TNFa* | Tumor necrosis factor alpha |
| *TNFb* | Tumor necrosis factor beta |
| *TNF RI* | TNFRSF1A , Tumor necrosis factor receptor superfamily member 1A |
| *TNF RII* | TNFRSF1B, Tumor necrosis factor receptor superfamily member 1B |

**Table S4. Results for the Se-modified reduced graphene oxide (RGO-Se) model calculated at the B3LYP/6-31G* level, gas phase//water**

| Spin | E_0_, A.U. | E_0_+ZPE, A.U. | ΔE,  kcal/  mol | E(HOMO/  LUMO), A.U./eV | ΔE_H/L_,  eV | D |
| --- | --- | --- | --- | --- | --- | --- |
| ^1^A | -6898.250839//  -6898.279101 | -6897.785481//  -6897.813981 | 0.0//  0.0 | -0.17944/ -0.09972//  -0.18539/ -0.10781 | 2.17//  2.11 | 7.2432//  12.0903 |
| ^3^A | -6898.214219  <S^2^>= 2.0731//  -6898.244074  <S^2^>= 2.0734 | -6897.751643//  -6897.781751 | 22.98//  21.98 | -0.14470/ -0.09576  -0.18555/ -0.13407//  -0.15295/ -0.10397  -0.19735/ -0.14016 | 1.33  1.40//  1.33  1.56 | 7.2838//  11.8878 |

*Energy (E), Atomic Units (A.U.), Zero Point Vibrational Energy (ZPE), Highest Occupied Molecular Orbital (HOMO), Lowest Unoccupied Molecular Orbital (LUMO), Dipole Moment (D)*

**Equations**

Equations 1 and 2 were used to calculate the ionization potential *(IP)* and electron affinity *(EA)* values:

| *IP* = -𝐸_HOMO_ | **Equation 1** |
| --- | --- |
| *EA* = -𝐸_LUMO_ | **Equation 2** |

For global hardness (*η)* and global electronegativity (*X*) values Equations 3 and 4 were used:

| $\eta=\frac{[IP-EA]}{2}=-\frac{[E_{\mathrm{LUMO}}-E_{\mathrm{HOMO}}]}{2}$ | **Equation 3** |
| --- | --- |
| $X=\frac{[IP+EA]}{2}=-\frac{[E_{\mathrm{LUMO}}+E_{\mathrm{HOMO}}]}{2}$ | **Equation 4** |

Global electrophilicity (*ω* ) values were calculated by Equation 5:

| $\omega=\frac{\mu^{2}}{2\eta}$, | **Equation 5** |
| --- | --- |

where$\mu=\frac{E_{\mathrm{HOMO}}+E_{\mathrm{LUMO}}}{2}$is the chemical potential (*μ*) of the system.

The global softness (*σ* ) values were computed using Equation 6:

| $\sigma=\frac{1}{2\eta}$ | **Equation 6** |
| --- | --- |

The propensity to donate electron (*ω*-) was computed using Equation 7:

*ω*- = (𝐸_LUMO_)^2^/(2*η*) **Equation 7**

The propensity to accept electron (*ω*+) was computed using Equation 8:

*ω*+ = (𝐸_HOMO_)^2^/(2*η*) **Equation 8**

*Highest Occupied Molecular Orbital (HOMO), Lowest Unoccupied Molecular Orbital (LUMO)*
